# Combined Systems Approaches Reveal a Multistage Mode of Action of a Marine Antimicrobial Peptide against Pathogenic Escherichia coli and Its Protective Effect against Bacterial Peritonitis and Endotoxemia

**DOI:** 10.1128/AAC.01056-16

**Published:** 2016-12-27

**Authors:** Xiumin Wang, Da Teng, Ruoyu Mao, Na Yang, Ya Hao, Jianhua Wang

**Affiliations:** a Key Laboratory of Feed Biotechnology, Ministry of Agriculture, Beijing, China; b Gene Engineering Laboratory, Feed Research Institute, Chinese Academy of Agricultural Sciences, Beijing, China

**Keywords:** marine antimicrobial peptides, N4, mechanism, Escherichia coli, lipopolysaccharide

## Abstract

A marine arenicin-3 derivative, N4, displayed potent antibacterial activity against Gram-negative bacteria, but its antibacterial mode of action remains elusive. The mechanism of action of N4 against pathogenic Escherichia coli was first researched by combined cytological and transcriptomic techniques in this study. The N4 peptide permeabilized the outer membrane within 1 min, disrupted the plasma membrane after 0.5 h, and localized in the cytoplasm within 5 min. Gel retardation and circular dichroism (CD) spectrum analyses demonstrated that N4 bound specifically to DNA and disrupted the DNA conformation from the B type to the C type. N4 inhibited 21.1% of the DNA and 20.6% of the RNA synthesis within 15 min. Several hallmarks of apoptosis-like cell death were exhibited by N4-induced E. coli, such as cell cycle arrest in the replication (R) and division(D) phases, reactive oxygen species production, depolarization of the plasma membrane potential, and chromatin condensation within 0.5 h. Deformed cell morphology, disappearance of the plasma membrane, leakage of the contents, and ghost cell formation were demonstrated by transmission electron microscopy, and nearly 100% of the bacteria were killed by N4. A total of 428 to 663 differentially expressed genes are involved in the response to N4, which are associated mainly with membrane biogenesis (53.9% to 56.7%) and DNA binding (13.3% to 14.9%). N4-protected mice that were lethally challenged with lipopolysaccharide (LPS) exhibited reduced levels of interleukin-6 (IL-6), IL-1β, and tumor necrosis factor alpha (TNF-α) in serum and protected the lungs from LPS-induced injury. These data facilitate an enhanced understanding of the mechanisms of marine antimicrobial peptides (AMPs) against Gram-negative bacteria and provide guidelines in developing and applying novel multitarget AMPs in the field of unlimited marine resources as therapeutics.

## INTRODUCTION

Pathogenic Escherichia coli not only can cause diarrheal disease in animals and human beings but also can lead to human urinary tract infection, meningitis, and pneumonia (1, 2). Although these diseases are effectively controlled by current antibiotics, resistance to these antibiotics is on the rise (1). Meanwhile, antibiotic application also indirectly results in the release of lipopolysaccharide (LPS), which is the major component of the outer membrane of Gram-negative bacteria and induces a series of diseases such as severe sepsis, septic shock, and systemic inflammatory response syndrome (3, 4). However, to date, no therapeutic agents have been shown to be efficacious enough to treat these LPS-induced diseases. Removal of Gram-negative bacteria by antimicrobial peptides (AMPs) may be an effective strategy to prevent LPS-induced pathophysiological responses (4, 5).

A total of 2,684 natural and synthetic AMPs, which are potential antibacterial agents, have been currently registered in the Antimicrobial Peptide Database (http://aps.unmc.edu/AP/main.php), but less than 5% of them are from marine resources (6). Marine AMPs have properties of biomedical importance, immunomodulatory activities, and signal transduction capability in mammalian hosts, which make them attractive templates for designing new drugs and pharmaceuticals (6). Meanwhile, due to their inherent ability to sustain activity under high salt concentrations, marine AMPs may have a high probability of success in *in vivo* systems and can be further regarded for clinical trials (6). Arenicin-3, a novel member of the arenicin family from the marine lugworm Arenicola marina with two disulfide bonds (Cys3-Cys20 and Cys7-Cys16) and four positive charges, was shown to form a 21-residue amphipathic β-sheet structure. In addition, it has higher activity than arenicin-1 and arenicin-2 *in vitro* against a variety of Gram-negative bacteria. However, the arenicin-3 molecule showed very high protein binding to serum components. A variant of arenicin-3 (5Y-5N, 17Y-17H), NZ17074, abbreviated as N4, which is undergoing preclinical studies, has a lower serum-binding ability and higher activity against Gram-negative bacteria, including resistant strains of E. coli and fungi, than its parent (7). Among members of the arenicin family, the mechanism of action, which includes binding to, intercalation into, and permeabilization of the model membranes, has been investigated for arenicin-1 against E. coli strain WBB01 (8). Arenicin-2 forms dimers by parallel association of the C-terminal strands and packs in higher-order aggregates by the loose parallel association of the N-terminal strands with the anionic lipid head groups (in addition, there is a possibility of intercalation between them) (9). However, the killing mechanism of N4 against Gram-negative bacteria is not yet elucidated and some AMPs exhibit remarkable specificity for particular AMP-bacterium pairings (10). Moreover, these physical consequences of arenicin interactions with cell membranes provided only circumstantial evidence as to the mechanism of action and did not account for their lethal activities or the remarkable specificities of their actions against bacteria.

In recent years, genome-wide transcriptional responses to challenges with antimicrobial agents have been developed as a source of information on the mode of action of an agent (11). Hong et al. demonstrated that the transcript levels of 26 genes changed significantly following treatment with α-helical cecropin A using whole-genome microarrays; only some genes such as *csgD* and *yiaT* are likely to encode membrane proteins, whereas 42% of the transcripts corresponded to protein products with unknown functions (12). Nielsen et al. found that arenicin-3 led to decreased expression of translation, translation factors (*rpl*, *rps*, and *rpm*), and phage shock protein-encoding genes (*pspA*, *pspB*, *pspC*, and *pspD*) and increased expression of lipoprotein (*osmB*) and regulon (*soxS* and *bdm*) by genomic sequencing and microarray analysis. Moreover, they found that *mlaC* single nucleotide polymorphism (SNP) provided arenicin-3 resistance in E. coli (13). However, these transcriptomic results were not connected with the antibacterial mode of action of the AMPs. Recently, Kozlowska found that the combined systems approach of cytology, metabolomes, and transcriptomics could accurately predict the mode of action of AMPs against E. coli NCTC 9001, which provided a fresh perspective for mechanism studies (14).

The goals of the present study were to investigate the antibacterial mechanism of N4 against pathogenic E. coli CVCC195 and to examine its potential applications. We conducted a comprehensive study on the mode of action of N4 against E. coli via a series of cell biology assays that included binding to LPS, permeabilization of the membranes, insertion into DNA, and induction of apoptosis-like cell death. To further elucidate the expression of specific genes correlated to the mechanism of action of N4, the global gene expression of E. coli in the presence of N4 was also analyzed by RNA sequencing. In addition, the antibacterial and detoxifying activity of N4 was evaluated in mice challenged with E. coli and its LPS, respectively.

## RESULTS

### N4 forms an amphipathic β-sheet.

**FIG 1:**
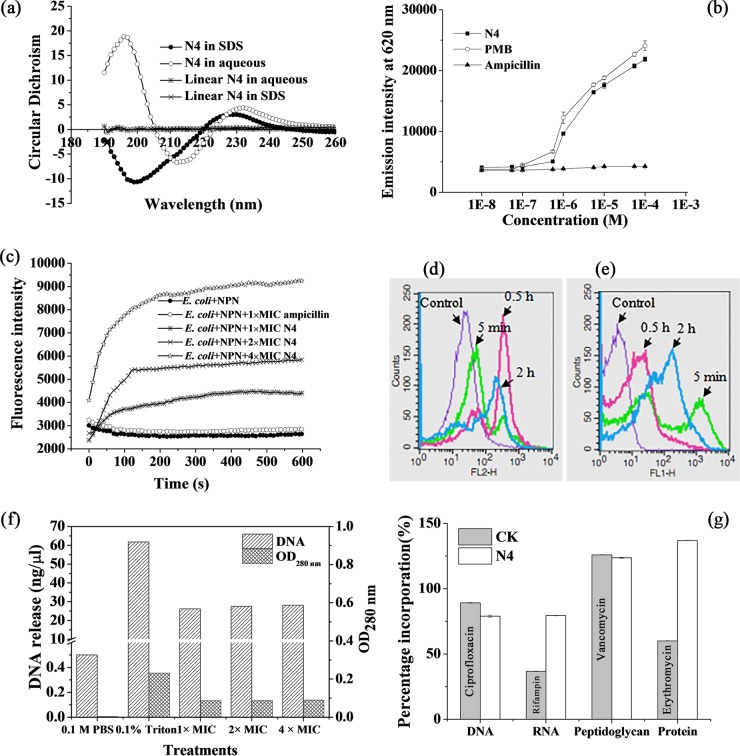
Structural analysis of N4 and its effects on LPS, cell membrane, and macromolecular biosynthesis in E. coli. (a) CD spectrum analysis of the second structure of N4 in an aqueous or SDS solution. (b) Displacement of LPS-bound BC by N4. Ampicillin and PMB were used as negative and positive controls, respectively. (c) Outer membrane permeabilization kinetics of E. coli cells treated with N4. The cells (10^8^ CFU/ml) were incubated with 1 mM NPN before the addition of 1×, 2×, and 4× MIC N4. The fluorescence of NPN was monitored for 10 min. (d and e) Flow cytometric analysis of the changes in membrane permeability. E. coli cells (10^8^ CFU/ml) were incubated with 1× MIC N4 (d) or FITC-labeled N4 (e) for 5 min, 0.5 h, and 2 h. The control cells had no peptide treatment. The bacterial cells were treated with N4, and the percentages of PI-permeable cells were 0.76% (control), 15.71% (5 min), 62.42% (0.5 h), and 32.29% (2 h) (d). The bacterial cells were treated with FITC-labeled N4, and the percentages of positive cells were 0% (control), 45.95% (5 min), 44.68% (0.5 h), and 69.19% (2 h) (e). (f) Efflux of DNA from E. coli induced by N4. The bacterial cells (10^6^ CFU/ml) were treated with 1×, 2×, and 4× MIC N4 at 37°C for 1 h. The amount of DNA was measured with an UV spectrophotometer. The cells treated with 0.1 M phosphate-buffered saline (PBS) and 0.1% Triton X-100 were used as the negative and positive controls, respectively. (g) Effects of N4 on the macromolecular biosynthesis in E. coli. Ciprofloxacin (8× MIC), rifampin (4× MIC), vancomycin (2× MIC), and erythromycin (2× MIC) were used as controls (CK) (gray bars). The experiment was repeated in triplicate.

The surfactant sodium dodecyl sulfate (SDS) provides a hydrophobic environment for polypeptides and promotes the stabilization of peptide conformation through hydrophobic interactions between peptides and SDS. As shown in Fig. 1a, in the absence of SDS, the secondary structure of N4 was predominantly characterized by β-sheet (86.9%) with a characteristic positive maximum at 230 nm and a negative minimum at 200 nm. However, N4 was induced into a distinct α-helical structure (61.1%) in 10 mM SDS. No folded structures were observed for the linear N4 in the aqueous solution and SDS. N4 may experience some untwisting upon formation of a β-structural pore and form an α-helix, which may be associated with its membrane-directed activity (15).

### N4 displayed potent antibacterial activity, low cytotoxicity, and no resistance.

Significant antibacterial activity of N4 against Escherichia and Salmonella was observed, with MICs from 0.25 to 1 μg/ml and minimal bactericidal concentrations (MBCs) ranging from 0.5 to 1 μg/ml (Table 1). Against Pseudomonas, MICs and MBCs of N4 ranged from 2 to 16 μg/ml. The MIC and MBC values of N4 against Staphylococcus aureus and Candida albicans were relatively high (16 to 32 μg/ml). N4 did not show activity against Listeria ivanovii, Streptococcus suis, Enterococcus faecium, Bacillus licheniformis, and Saccharomyces cerevisiae up to 16 μg/ml.

**TABLE 1:** MIC and MBC values of N4 against bacteria and fungi

Strain	N4 MIC (μg/ml)	N4 MBC (μg/ml)
Gram-negative bacteria		
E. coli CVCC195	0.5–1	1
E. coli CVCC1515	0.25	0.5
E. coli O157	0.5	1
E. coli CMCC44102	1	1
E. coli ER2566	0.5	0.5
Salmonella enterica serovar Enteritidis CVCC3377	0.25	0.5
S. enterica serovar Pullorum CVCC1789	0.25	0.5
*S*. Pullorum CVCC1802	0.25	0.5
S. enterica serovar Choleraesuis CVCC3380	0.25–0.5	0.5
*S*. Pullorum CVCC503	0.25	0.5
Pseudomonas aeruginosa CVCC2087	4	8
P. aeruginosa CMCC10104	2	4
P. aeruginosa ATCC 10145	4	16
P. aeruginosa ATCC 15442	4	8
P. aeruginosa ATCC 27853	4–8	16
Gram-positive bacteria		
Staphylococcus aureus ATCC 6538	16	16
S. aureus ATCC 43300	16	32
S. aureus ATCC 25923	0.5–1	NA^*a*^
Staphylococcus epidermidis ATCC 26069	8	NA
Listeria ivanovii ATCC 19119	>16	>16
Streptococcus suis CVCC3309	>16	>16
Clostridium perfringens CVCC61	32	NA
C. perfringens CVCC46	>32	NA
Enterococcus faecium CMCC1.2136	>16	>16
Bacillus licheniformis CMCC1.265	>16	>16
Bacillus subtilis DSM5750	2	NA
B. licheniformis DSM5749	4	NA
Fungi		
Candida albicans CMCC2.2411	16	16
Candida utilis CMCC2.1180	16	NA
Saccharomyces cerevisiae CMCC2.1546	>16	>16
Pichia pastoris X-33	16	NA

a NA, no detection.

The 3-(4,5-dimethyl-2-thiazolyl)-2,5-diphenyl-2H-tetrazolium bromide (MTT) assay results showed that only 1.2% of porcine intestinal epithelial cells were inhibited by N4 at 1 μg/ml. A cell inhibition of 8.0% to 13.0% was observed in a range of 2 to 16 μg/ml of N4. Higher percentages of cell inhibition ranging from 20.6% to 37.9% were observed from 32 μg/ml to 128 μg/ml of N4 (data not shown). For mouse peritoneal macrophages, in the concentration range of 1 to 8 μg/ml of N4, peptide exerted moderate cytotoxicity (≤9.2%). At higher concentrations of 16 to 128 μg/ml, a significant increase in cytotoxicity was observed, with inhibition ratios of 12.1% to 48.0% (see Table S1 in the supplemental material).

After 15 serial passages in the presence of N4, the MICs did not change, which indicated that no mutants of E. coli resistant to N4 were produced (data not shown). These features of N4 indicate that it is a good candidate for the development of novel antibiotic agents from marine sources.

### N4 bound to LPS.

To test whether N4 binds to E. coli LPS, the MIC values were determined and 5-((4-(4,4-difluoro-5-(2-thienyl)-4-bora-3a,4a-diaza-s-indacene-3-yl)phenoxy)acetyl)amino)pentylamine, hydrochloride (BODIPY-TR-cadaverine, or BC) probe displacement methods were performed. The MIC values of N4 and its LPS were 0.5 to 1 and 2 μg/ml, respectively (data not shown), but LPS did not exhibit antibacterial activity against E. coli (MIC > 64 μg/ml). N4 treated with LPS displayed a 1- to 3-fold decrease of antimicrobial activity against E. coli compared to N4, which confirmed that N4 could bind to LPS.

Similar to polymyxin B (PMB), N4 induced a dose-dependent displacement of BC (Fig. 1b). As expected, ampicillin, which binds to penicillin binding proteins, did not displace BC from its binding to LPS. This result indicated that N4 may interact with lipid A of LPS via interactions similar to those of BC.

### N4 disrupted the membrane of E. coli cells. (i) Permeabilization of the outer membrane within 1 min.

The outer membrane, a unique asymmetric lipid bilayer composed of LPS in the outer leaflet and phospholipid in the inner leaflet, is a very important cellular structure of Gram-negative bacteria and serves as a selective permeation barrier (16). N-phenyl-1-naphthylamine (NPN) is a hydrophobic fluorescent probe that emits weak fluorescence in an aqueous environment and strong fluorescence when incorporated into the hydrophobic core of a bacterial cell membrane (17). The fluorescence from E. coli cells was monitored after incubation with N4 and NPN. As shown in Fig. 1c, N4 induced a time-dependent and concentration-dependent NPN fluorescence increase in intact E. coli cells, which suggests that N4 could instantly (within 1 min) permeabilize the outer membrane of intact E. coli cells. Higher concentrations induce a stronger NPN uptake as shown by the stronger fluorescence that was observed and indicating that N4 made the outer membrane more permeable.

### (ii) Disruption of the plasma membrane after 0.5 h of treatment.

The plasma membrane, which is composed of two layers of phospholipids and embedded with proteins, is a thin semipermeable membrane layer that plays a vital role in protecting the integrity of the cell interior (18). The effect on membrane permeability was evaluated using propidium iodide (PI), a cationic nucleic acid dye that is excluded by viable cells with intact membranes, whereas it enters cells with damaged membranes and binds to DNA or RNA. The fluorescence conferred by PI indicates the degree of cell damage, cell permeability, and ultimately, cell death (19). Figure 1d shows that 1× MIC N4 induced the influx of PI, which is indicative of cell membrane permeabilization. A progressive increase in cell fluorescence from PI occurred because of the elevated entry of the dye into the cells. The percentages of PI-permeable E. coli cells treated with N4 for 5 min, 0.5 h, and 2 h were 15.71%, 62.42%, and 32.29%, respectively, which are much higher than that of the untreated cells (0.76%). Membrane permeabilization may occur concomitantly with the loss of cell viability, which suggests that N4 permeabilized the plasma membrane and entered the cell within 5 min, and the plasma membrane of E. coli was disrupted after 0.5 h of treatment with N4, which may be a lethal event in peptide action (20).

The fluorescence-activated cell sorter (FACS) analysis of the cells incubated with fluorescein isothiocyanate (FITC)-labeled N4 demonstrated that the fluorescence intensity of the treated cells increased as the treatment time was prolonged (Fig. 1e). The percentages of the permeable E. coli cells treated with 1× MIC FITC-labeled N4 were 45.95%, 44.68%, and 69.19% for 5 min, 0.5 h, and 2 h, respectively, higher than that of the control cells (0%), and this indicated that FITC-labeled N4 could enter E. coli cells within 5 min. This result suggests that N4 has antibacterial and channel-forming properties, which is closely related to an antiparallel β-sheet configuration contained in peptide (21).

The integrity of the plasma membrane was further examined by monitoring the amount of DNA released from the cells treated with N4 for 1 h. As shown in Fig. 1f, the DNA contents of the E. coli treated with 1×, 2×, and 4× N4 were 26.2, 27.5, and 28.1 ng/μl, respectively, slightly lower than DNA contents of the cells treated with 0.1% Triton X-100 (61.8 ng/μl). These results indicate that the plasma membrane is one target site for N4 and interaction of N4 with the bacterial membrane forms transient pores or channels after 1 h of treatment, which leads to the leakage of the cell contents and cell death (22).

The action of N4 against E. coli indicated that the permeabilization of the outer and plasma membranes of viable E. coli occurred within 1 min and 5 min, respectively, and that the plasma membrane of E. coli was damaged after 0.5 h of treatment with N4.

### N4 inhibited the synthesis of DNA and RNA precursors within 15 min.

The incorporation of radioactive precursors into DNA ([^3^H]thymidine), RNA ([^3^H]uridine), protein ([^3^H]leucine), and peptidoglycan ([^3^H]glucosamine) was measured to evaluate the effects of N4 on macromolecular synthesis in E. coli CVCC195. As shown in Fig. 1g, a significant inhibition of [^3^H]thymidine (21.1%) and [^3^H]uridine (20.6%) incorporation was observed at 15 min of exposure of E. coli to 1× MIC N4. In addition, N4 induced an increase in protein and peptidoglycan, which suggested that N4 is a DNA and RNA synthesis inhibitor. This effect of N4 was similar to that observed for indolicidin (23). The antimicrobial activity of N4 is most likely due to the inhibition of DNA and RNA synthesis as a result of the binding of N4 to cellular DNA and RNA.

### N4 specifically bound to DNA and changed the DNA conformation. (i) Gel retardation.

**FIG 2:**
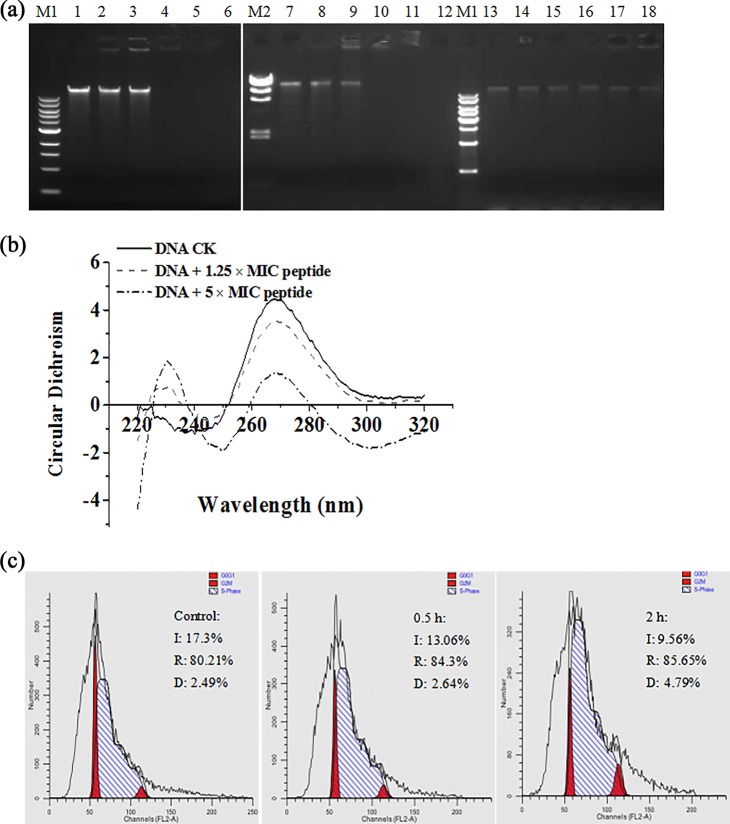
Binding of N4 to E. coli DNA and effects of N4 on the cell cycle of E. coli. (a) Gel retardation analysis of the binding of N4 to DNA. M1, 1,000-kb DNA ladder (Tiangen) (left panel); M2, λ hindIII; M1, 1,000-kb DNA ladder (TransGen Biotech) (right panel); lanes 1 to 6, the mass ratios of N4 and genomic DNA from E. coli were 0, 0.25, 0.5, 1, 2.5, and 5; lanes 7 to 12, the mass ratios of N4 and genomic DNA from Salmonella sp. CVCC3377 were 0, 0.25, 0.5, 1, 2.5, and 5; lanes 13 to 18, the mass ratios of N4 and genomic DNA from S. aureus ATCC 6538 were 0, 0.25, 0.5, 1, 2.5, and 5. (b) CD spectra of E. coli DNA in the presence of increasing amounts of N4. The mass ratios of N4 and genomic DNA from E. coli were 0, 1.25, and 5. (c) Effects of N4 on the cell cycle of E. coli. E. coli (10^8^ CFU/ml) was cultured alone as a control group (left) or cultured in the presence of 1× MIC N4 for 0.5 h (middle) or 2 h (right). The cell cycle distribution was determined by the PI staining method and analyzed by flow cytometry.

In an attempt to seek intracellular targets, the DNA-binding ability of N4 was evaluated by DNA gel retardation (Fig. 2a). At a peptide/DNA mass ratio of 0.5, nearly all of the genome DNA from E. coli and Salmonella sp. strain CVCC3377 was still able to migrate into the gel in the same way as noncomplexed DNA. At mass ratios of 1, 2.5, and 5, no DNA bands were detected on the gel for E. coli and Salmonella sp. CVCC3377, which showed the intrinsic DNA-binding ability of N4. This is consistent with a previous report that MDpep9 from the edible larvae of houseflies could bind to genomic DNA from E. coli (24). S. aureus ATCC 25923 DNA migrated normally in the gel as noncomplexed DNA and remained unbound at a lower mass ratio of 0.25, which indicated that N4 did not involve DNA binding. This DNA-binding specificity result is consistent with that of the antimicrobial spectrum of N4, exhibiting specific and selective bactericidal activity.

### (ii) CD spectra.

CD spectrum analysis is an extraordinarily sensitive and useful technique to monitor changes in DNA morphology during drug-DNA interactions (25). The DNA-binding affinity of N4 was further assessed using a CD spectrometer. As shown in Fig. 2b, the B-DNA structure was observed in the absence of N4, which was characterized by a positive long-wavelength band at 270 nm and a negative band at approximately 240 nm. At a low N4-to-DNA ratio of 1.25, the DNA spectrum was characterized by a dominant negative band at 245 nm, due to the right-handed helicity B form of DNA and a positive band at 270 nm, due to the base stacking between N4 and DNA bases; there was a slight additional reduction in the CD amplitude, which indicated that N4 has an effect on the helicity structure of the DNA (26). The DNA CD spectra changed at a higher N4-to-DNA ratio of 5.0, which contained a negative band at approximately 250 nm and a positive band at approximately 270 nm, and there was a greater decrease in the CD amplitude. These observations suggest that DNA binding of N4 induced certain conformational changes from the B- to C-like conformation within the DNA molecule and unwound DNA base pairs with destabilization of the DNA double helix (27, 28). It is possible that N4 could intercalate into the base pairs in a helix of DNA or locate in the hydrophobic environment of DNA, and the complex could be stabilized by the stacking interaction with the DNA bases (28). This CD result is in agreement with the conclusion of the above-described gel retardation assay, which indicated that DNA binding with N4 may inhibit the macromolecular synthesis needed for the life cycle of bacterial cells.

### Hallmarks of apoptosis-like cell death were exhibited by N4-induced E. coli. (i) Cell cycle arrest within 0.5 h to 2 h.

DNA damage triggers a series of carefully controlled processes that stop cell cycle progression to ensure that cell division will not proceed to the next phase and leads to cell cycle arrest in either phase (27). As shown in Fig. 2c, the percentages of the control cells in the initiation (I), replication (R), and division (D) phases were 17.3%, 80.21%, and 2.49%, respectively. Exposure to 1× MIC N4 for 0.5 h and 2 h resulted in an increase in the percentage of R- and D-phase cells in a time-dependent manner (from 84.3% to 85.65% and from 2.64% to 4.79%, respectively) and a corresponding reduction in the percentage of cells in the I phase (from 13.06% to 9.56%) (Fig. 2d and e). The results show that the antibacterial action of N4 was accompanied by an increase in the percentage of R- and D-phase cells, which is a typical cell cycle arrest and one of the typical markers of apoptosis-like cell death. The R/D cell cycle arrest induced by N4 within 0.5 h to 2 h is most likely the consequence of DNA damage (27). This indicated that N4 inhibited the replication of DNA and the division of cells within 0.5 h to 2 h.

### (ii) Induction of intracellular ROS production within 0.5 h.

**FIG 3:**
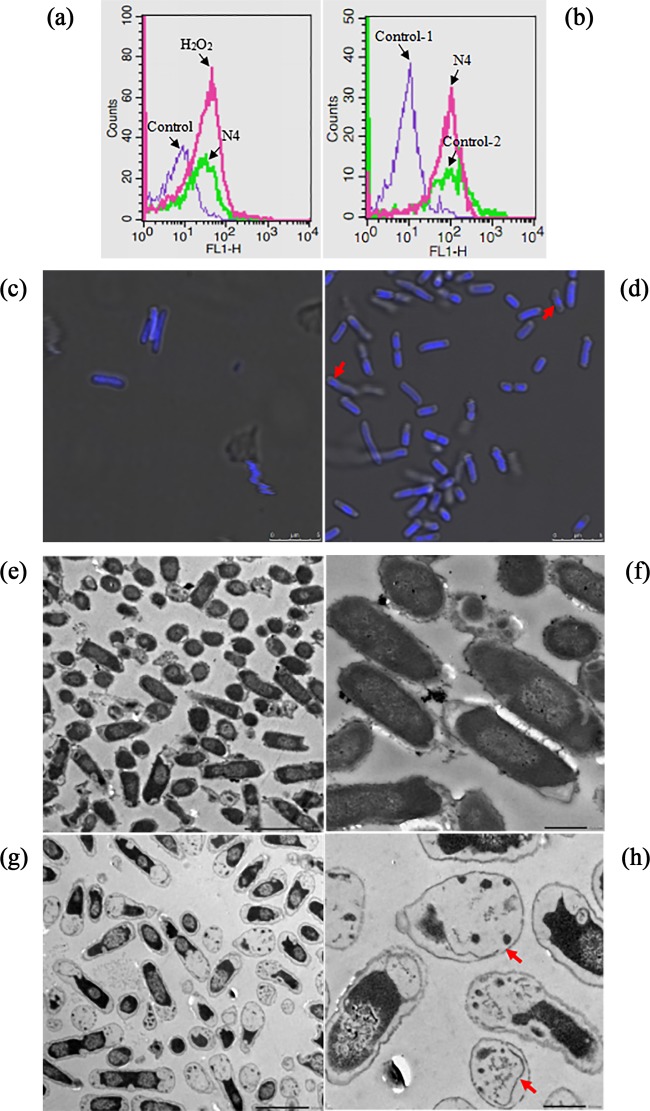
Apoptosis-like cell death of E. coli induced by N4. (a) Intracellular ROS accumulation in E. coli cells treated with N4. E. coli cells (10^8^ CFU/ml) were treated with 1× MIC N4 for 0.5 h, stained with DHR-123, and analyzed on a flow cytometer. (b) Plasma membrane depolarization in E. coli cells treated with N4. E. coli cells (10^8^ CFU/ml) were treated with 1× MIC N4 for 1 h, stained with RH-123, and analyzed on a flow cytometer. Control-1, healthy cells (without treatment), no staining with RH-123; Control-2, healthy cells stained with RH-123. (c and d) Effects of N4 on chromatin condensation in E. coli assayed using DAPI staining. E. coli cells (10^8^ CFU/ml) were treated with 1× MIC N4 (d) or without peptide (c) for 0.5 h and stained with DAPI. Arrowheads indicate condensation of chromatin. (e to h) TEM images of E. coli in the absence of N4 or in the presence of 4× MIC N4 for 2 h. (e and f) Untreated cells (enlarged view in panel f). (g and h) Cells treated with N4 (enlarged view in panel h). Arrowheads indicate the disappearance of the plasma membrane and ghost cells.

Several AMPs, such as LL-37 and CM15, have been reported to induce the production of reactive oxygen species (ROS), which causes oxidative stress damage (29, 30). To find the underlying mechanism of N4-induced apoptosis-like cell death, ROS production, a major cause of apoptosis, can be monitored using dihydrorhodamine-123 (DHR-123) (31). As shown in Fig. 3a, the E. coli cells treated with 1× MIC N4 for 0.5 h displayed high ROS levels compared to the untreated cells. There was a significant increase in fluorescence when the cells were treated with 2.5 mM H_2_O_2_. This result showed that N4 promoted the generation of ROS (via a common metabolic mechanism), which are crucial apoptotic regulators and have destructive actions on both DNA and proteins (27).

### (iii) Depolarization of the plasma membrane within 1 h.

Changes in the transmembrane potential have been considered a hallmark of apoptosis (32). The changes in plasma membrane potential (ΔΨ) were examined using rhodamine-123 (RH-123), which accumulates on the inner surface of intact membranes. As shown in Fig. 3b, the low levels of fluorescence from the unstained cells without treatment (Control-1) were observed because of cellular autofluorescence. The E. coli cells treated with 1× MIC N4 for 1 h had markedly reduced cellular fluorescence compared to that of the stained cells without treatment (Control-2). This was similar to the membrane depolarization that occurred in E. coli cells treated with magainin 2 (33). This result confirmed that N4 traversed the outer membrane and caused potential plasma membrane depolarization, which leads to the loss of the proton gradient, the leakage of essential molecules, such as DNA, and cell death (34).

### (iv) Chromatin condensation within 0.5 h.

Chromatin condensation is a well-established cytological hallmark of apoptosis (22). To monitor the structural state of the bacterial chromatin after the peptide treatment, a DNA-specific and conformation-sensitive DAPI (4′,6-diamidino-2-phenylindole) dye was used. The untreated E. coli cells exhibited light staining (Fig. 3c), which was expected given the known fluorescence properties of the dye. After 0.5 h of treatment with 1× MIC N4, we observed highly ordered and focused, yet dim, chromatin staining in the cells, which indicated that in a fashion similar to that of LL-37, N4 migrates to the nucleus through the intact cell (35). The focal points were observed in a minority of the intact cells (approximately 10%), which indicated that localized condensation of chromatin material occurred and the DNA was seriously damaged by N4 (Fig. 3d). A similar change in DNA morphology was observed in E. coli treated with magainin as well (33).

### N4 induced extensive cellular damage and led to cell death of E. coli.

To gain additional direct insight into the interaction of N4 with E. coli, transmission electron microscopy (TEM) was performed on the bacterial cells treated with 4× MIC N4 for 2 h. The untreated E. coli cells were shaped and displayed normally with no damage to the structure of the plasma membrane or the outer membrane, and the cytoplasm appeared to have homogeneous electron density (Fig. 3e and f). As shown in Fig. 3g and 3h, cell swelling, cell disruption, plasmolysis, and partial disappearance of the plasma membrane were observed. Under these conditions, nearly 100% of bacteria were killed. The cytoplasm displayed a heterogeneous electron density, and the morphology of the cells was deformed. In agreement with the above-described membrane interaction results, gross leakage of the cellular cytoplasmic contents was observed with resultant ghost cell formation, which indicated that N4 caused cell death and induced lysis.

### Transcriptional profiles of E. coli treated with N4.

**FIG 4:**
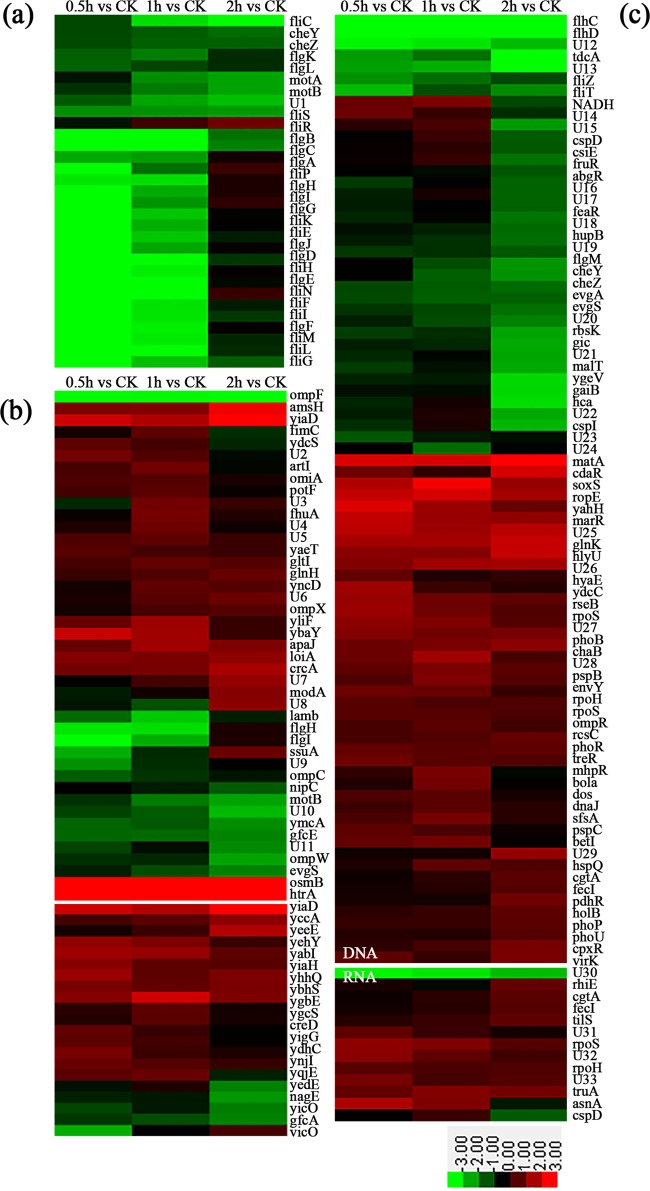
Identification of the genes of E. coli responsive to N4. The genes included here show significant differences in gene expression (FDR ≤ 0.001, fold change ≥ 2). The genes shown in red had upregulated expression, and those shown in green had downregulated expression in E. coli treated with N4 for 0.5 h, 1 h, and 2 h. Shown are a cluster enriched in flagellum-associated genes (a), one enriched in membrane-associated genes (b), and one enriched in DNA- and RNA-associated genes (c).

To further clarify the molecular mechanism of action, the global transcriptional response of E. coli to N4 was performed. In contrast to the untreated controls, N4-treated E. coli had 63 membrane-associated genes (see Table S2 in the supplemental material), 30 flagellum-associated genes (see Table S3 in the supplemental material), and 81 DNA-associated genes (see Table S4 in the supplemental material) with expression levels that were significantly changed after treatment for 0.5 to 2 h (Fig. 4) (false discovery rate [FDR] ≤ 0.001, |log_2_ ratio| ≥1). These data suggest that the main influence of N4 on E. coli cells was disruption, loss, or disorganization of the membrane and the genomic DNA from the bacteria.

### Membrane-associated genes.

Flagella are essential membrane structures for the pathogenic potential of bacteria and mediate secretion of extracellular toxins, and nearly 50 genes are involved in flagellar formation and function in E. coli (36). The transcription of more than 40 flagellar genes is hierarchically controlled by environmental conditions via the master regulator operon *flhDC* (37). Among these membrane-associated genes, nine flagellar assembly genes (*fliP*, *fliQ*, *fliG*, *fliI*, *fjiJ*, *fliN*, *flhA*, *flhB*, and *flgH*) had expression that was significantly decreased 2.1- to 10-fold; another 22 flagellar genes were strongly downregulated after treatment for 0.5 h or 1 h but not detected after treatment with N4 for 2 h, which inhibited flagellum biosynthesis (Fig. 4a; see also Fig. S1 and Table S3 in the supplemental material). Several genes, such as *fliP* and *fliQ* encoding membrane proteins, are predominantly located in the membrane fraction, which indicated that N4 may impair the bacterial membrane structures (38). It was concluded that one of the major transcriptional responses of E. coli to N4 was the downregulation of flagellar genes. The decrease in flagellar gene expression permits bacteria to block proton influx through both the H^+^/ATPase and the flagellar base structure, which affects vital ATP synthesis, results in impairment of swimming and swarming motility, and further influences the pathogenesis of bacteria (39).

Downregulation of chemotaxis gene expression is another major transcriptional response in E. coli upon treatment with N4. Genes encoding the chemotaxis protein CheR/B in E. coli were upregulated at 0.5 h and 1 h and were then downregulated at 2 h by treatment with N4. Another 11 chemotaxis genes (*cheA*, *cheW*, *mcp*, *cheZ*, *cheY*, *motA*, *tsr*, *tar*, *malE*, *tap*, and *aer* but not *dppA* or *motB*) had an expression that was significantly decreased 2- to 5-fold at 0.5 to 2 h after the N4 treatment compared with the control (see Fig. S2 in the supplemental material), which induced a change in membrane potential during bacterial chemotaxis. Bacterial chemotaxis is the process by which bacteria efficiently and rapidly sense changes in their chemical environment and move to more favorable conditions (37).

The expression level of *ompC* and *ompF* genes encoding the major outer membrane pore proteins was markedly decreased 1.1- to 15-fold after treatment with N4 for 0.5 to 2 h, which caused a membrane permeability change (Fig. 4b; see also Table S2 in the supplemental material). The expression level of *agaC*, *agaD*, and *agaF* genes encoding the phosphotransferase system (PTS), which is involved in sugar uptake, phosphorylation, and regulation of a number of other metabolic pathways, was also decreased 1- to 1.3-fold after N4 treatment for 0.5 to 2 h. Both AgaC and AgaD are integral constituents of the membrane, and their downregulation suggests that N4 caused cell membrane disruption, which destroys the transport of a large number of carbohydrates in bacteria (40).

The *mdtA*, *mdtB*, and *mdtC* genes, encoding multidrug transport proteins, were significantly upregulated 1.6- to 3.3-fold in the presence of N4, which conferred resistance to this peptide (41) (see Table S5 in the supplemental material), but this needs further study. Other genes (*phoR*, *rstB*, *degP*, *pagP*, *dppB*, *cls*, and *pstA*), involved in encoding the sensor histidine, serine kinase, permease, synthase, and transferase, were markedly upregulated 1- to 16.1-fold after N4 treatment (Fig. 4c; see also Table S5 in the supplemental material), wherein the sensor histidine RstB transmits stress signals to cytoplasmic response regulators to control the expression of sigma factors and then mediates other genes to respond to environmental changes (42).

The above-identified genes that are linked to membranes support the cytological result that N4 largely acted on the membrane of E. coli.

### DNA-associated genes.

Among DNA binding-associated genes, the *fliA*, *flhC*, and *flhD* genes of the flagellar RNA polymerase sigma factors and transcriptional activators were significantly downregulated 3.6- to 31-fold at 0.5 to 2 h after the N4 treatment compared with the control (Fig. 4c). One of the master regulators of intracellular E. coli gene expression is the PhoPQ two-component system, which affects the expression of several known virulence functions (43). The PhoP-PhoQ-activated (*pagP*) gene with an increase in the expression of 1.6- to 3-fold was identified as important for inducible AMP resistance and increased acylation of lipid A (43). In this study, expression of the *phoP* gene was upregulated 1.1-fold after treatment for 2 h with N4 (Fig. 4c), which resulted in outer membrane alterations that included modification of lipid A of LPS, which is the major cell surface molecule of Gram-negative bacteria (43).

The expression levels of *phoB*, *rstA*, *rcsA*, *rcsB*, *ompR*, *crxR*, and *holB* genes in the two-component system and metabolism were significantly elevated 1.1- to 11.9-fold, and the expression levels of the activating virulence genes *evgA* and *bvgA* and the two-component system, response regulator *yesN*, was downregulated 1.5- to 2.5-fold, which indicated that N4 reduces bacterial pathogenicity (Fig. 4c; see also Table S5 in the supplemental material). Compared with the treatment for 0.5 h and 1 h, 10 genes of the ATP-binding protein ribokinase transcriptional regulator (*phnC*, *aphB*, *yesN*, *rbsK*, *pilR*, *phoP*, *holB*, *crxR*, *glk*, and *evgA*) in E. coli treated with N4 for 2 h were uniquely expressed. The expression levels of *holB*, *phoP*, and *crxR* genes of DNA replication and AMP resistance were upregulated 1.1- to 1.6-fold, but *glk*, *evgA*, *phnC*, *aphB*, *yesN*, *rbsK*, and *pilR* genes had expression that was decreased 1- to 3.7-fold at 2 h after N4 treatment (Fig. 4c; see also Tables S4 and S5 in the supplemental material).

The expression levels of several cell cycle-associated genes, such as *dgcB*, *pleD*, and *rseP*, were upregulated 1.1- to 2.7-fold at 0.5 to 2 h after N4 treatment (see Fig. S4 and Table S5 in the supplemental material), which was in agreement with a previous result that PleD is a key regulator of cell cycle events and negatively regulates chemotaxis and motility during the G_1_ phase (38).

Moreover, several genes (*purE*, *purF*, *purM*, and *pyrD*) of the *pur* family, which are involved in purine or pyrimidine metabolism, were downregulated (data not shown). These genes take part in the conversion of phosphoribosyl pyrophosphate (PRPP) into the 5-formamidoimidazole-4-carboxamide ribotide (FAICAR), which can be converted into inosine monophosphate (IMP). This suggests that N4 may inhibit bacterial DNA replication or repair bacterial cell growth (44, 45).

DNA-associated changes in gene expression provide direct evidence that N4 can enter E. coli cells and affect the function of intracellular targets.

### N4 protected mice from a lethal challenge with E. coli or LPS. (i) E. coli-induced peritonitis.

**FIG 5:**
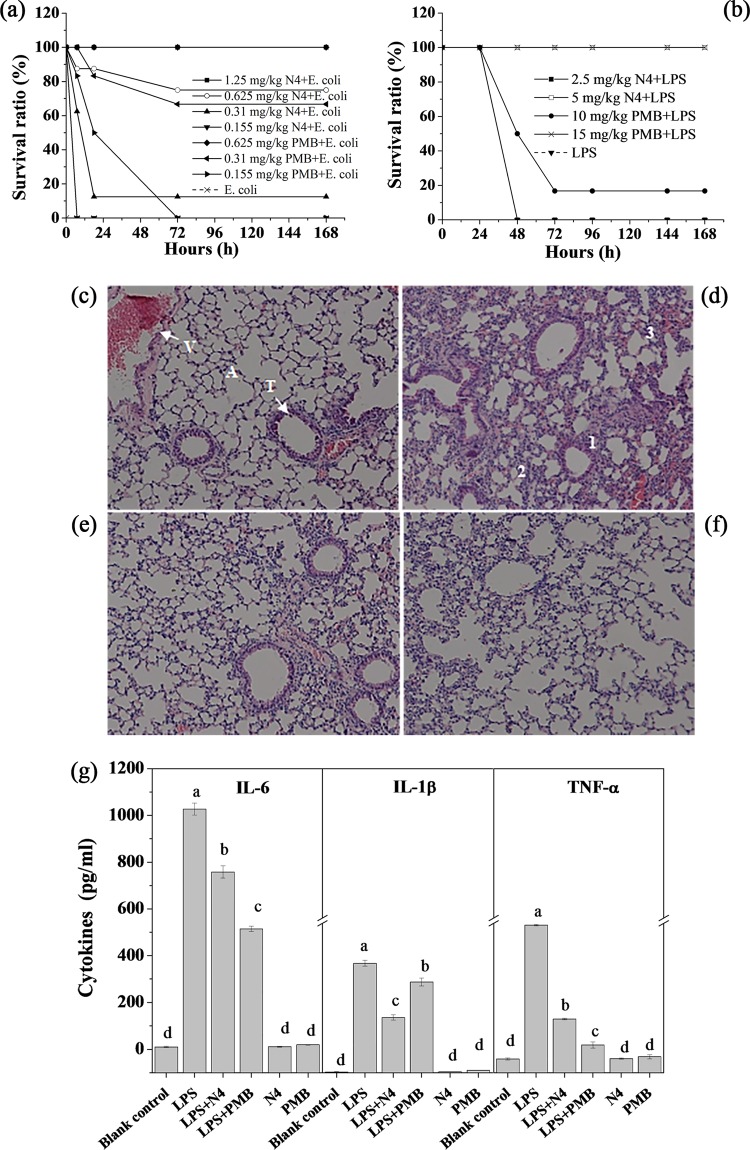
Protective efficacy of N4 in endotoxemic mice. Groups of four or eight mice were intraperitoneally injected with E. coli CVCC1515 (2.5 × 10^8^ CFU/ml, 1 ml) (a) or LPS from E. coli 0111:B4 (10 and 30 mg/kg of body weight) (b to g) followed by injection with PBS, PMB (10 and 15 mg/kg), and N4 (2.5 and 5 mg/kg). (a and b) Survival ratio of mice injected with E. coli CVCC1515 (a) or 30 mg/kg LPS (b) plus N4 or PMB. (c to f) Histological evidence of N4 on 30 mg/kg LPS-induced lung injury (original magnification, ×100). After sacrificing, the lung was fixed, embedded, and stained with hematoxylin and eosin. (c) Lung without LPS injection. V, vessel; A, alveoli; T, trachea. (d) Lung injected with LPS. 1, alveolar septum thickening; 2, pulmonary alveolar collapse; 3, inflammatory cell infiltration. (e) Lung injected with LPS and treated with N4 (5 mg/kg). (f) Lung injected with LPS and treated with PMB (15 mg/kg). (g) Inhibition of 10-mg/kg LPS-induced cytokine release in endotoxemic mice by N4. The blood was collected at 2 h or 8 h after challenge with LPS, and cytokines IL-6 (8 h), IL-1β (8 h), and TNF-α (2 h) were detected by ELISA kits. A different lowercase letter indicates a significant difference among the six treatments (*P* < 0.05).

Mice in the control group injected with PBS did not die throughout the experimental period (Fig. 5a). The mice without treatment began to die 12 h after inoculation with E. coli, and all of the mice were dead within 24 h. After treatment with 0.155, 0.31, 0.625, 1.25, and 2.5 mg of N4/kg of body weight, the survival rates of mice were 0, 12.5%, 87.5%, 100%, and 100%, respectively. The survival rates of mice treated with 0.155, 0.31, and 0.625 mg/kg PMB were 50%, 66.7%, and 100%, respectively (Fig. 5a).

### (ii) Intraperitoneal administration of LPS.

To evaluate the therapeutic activity of N4 in the endotoxemia model, mice were injected with N4 (2.5 and 5 mg/kg) or PMB (10 and 15 mg/kg). No mice that were injected twice with PBS died during the experimental period. The survival ratios of 5-mg/kg N4 and 15-mg/kg PMB treatment groups were 100%, which was higher than those of the negative-control group (0%), the 2.5-mg/kg N4 treatment group (0%), and the 10-mg/kg PMB treatment group (16.7%). This result indicated that N4 could protect mice from a lethal LPS challenge *in vivo* (Fig. 5b).

To explore if N4 can reduce lung injury from a lethal challenge with LPS, the lung damage degree was examined at 96 h after treatment. As shown in Fig. 5c, no pathological change was observed in the lung of mice injected with PBS, whereas mice injected with LPS plus PBS developed acute lung injury to a certain degree, and it was characterized by pulmonary alveolar collapse, alveolar septum thickening, and inflammatory cell infiltration (Fig. 5d). In contrast, the lungs of the mice injected with LPS plus N4 or PMB were apparently less damaged at 96 h (Fig. 5e and f). This suggests that similar to PMB, N4 protected the lung from damage by LPS in mice with endotoxemia.

To determine if the protective activity of N4 was associated with inflammatory cytokines, we measured the concentrations of interleukin-6 (IL-6), IL-1β, and tumor necrosis factor alpha (TNF-α) in the sera from mice with endotoxemia. As shown in Fig. 5g, the concentrations of IL-6, IL-1β, and TNF-α in sera of mice with endotoxemia treated with N4 (758.02, 147.69, and 143.78 pg/ml, respectively) or PMB (514.91, 242.82, and 74.03 pg/ml, respectively) were significantly lower than those of the LPS control group (1026.76, 292.87, and 394.89 pg/ml, respectively). This result indicated that similar to PMB, N4 inhibited the secretion of IL-6, IL-1β, and TNF-α in endotoxemic mice.

These data indicated that N4 could protect mice from lethal E. coli and an LPS challenge *in vivo*.

## DISCUSSION

There is a direct relationship between gene expression and a biophysical event in response to treatment with AMPs (14). The mechanism of the action of N4 against E. coli and antibacterial/detoxifying activity was systematically investigated by a combined approach for the first time in this study.

LPS is the molecular basis of the integrity of the outer membrane (3). It has been demonstrated that human cathelicidin can bind and inhibit LPS and exogenous murine cathelicidin can decrease TNF-α release (46). In our study, the BC probe displacement confirmed observations on the direct binding of N4 with LPS (Fig. 1b), which, in turn, possibly disrupted the interaction of LPS with its receptor Toll-like receptor 4 (TLR4) and led to inhibition of LPS-induced IL-6, IL-1β, and TNF-α release in mice (Fig. 5g) (47), thereby protecting them from LPS-induced damage to the lung (Fig. 5e). Moreover, N4 increased the survival ratio of peritonitis and endotoxemic mice and was found to be significantly superior to PMB in the endotoxemia model (Fig. 5a). Together, these results suggest that N4 is a potential antibacterial and endotoxemia therapeutic.

The addition of AMPs, such as arenicin-1 and melittin, to E. coli cells leads to cell death concomitant with intracellular K^+^ leakage and cell lysis (48, 49). This study indicated that N4 interacted with the E. coli membrane and caused NPN entrance into the outer membrane within 1 min (Fig. 1c), PI influx into the cells (Fig. 1d) within 5 min, and DNA efflux from the cells within 1 h (Fig. 1f). The TEM images further confirm the interaction of this peptide with the plasma membrane of E. coli, which resulted in the leakage of the intracellular contents (Fig. 3g and h). Meanwhile, the expression levels of the membrane-associated genes, such as *ompF*, *ompC*, *agaC*, and *agaD*, were significantly repressed after N4 treatment for 0.5 h (Fig. 4b), which indicated that membrane disruption occurred and that the target site of N4 was the E. coli cell membrane.

Another significant effect of N4 on E. coli was evident from its DNA-binding property. The genomic DNA from E. coli and Salmonella was completely inhibited by N4 to a DNA mass ratio higher than 1 with no visible inhibition in the migration of the S. aureus genomic DNA (Fig. 2a), which indicated that N4 selectively binds to bacterial DNA. The CD analyses indicated that N4 interacts with E. coli genomic DNA by insertion into the base pairs and changing the DNA conformation (Fig. 2b), which is similar to a previous report that peptide bound tightly to DNA. N4 inhibited DNA and RNA synthesis within 15 min within the cell by regulating transcriptional activator genes (*flhC*, *flhD*) and activating ATP-dependent RNA helicase (*rhlE* and *deaD*) (Fig. 1g and 4c) and disrupting the materials needed for the life cycle of bacteria (47).

**FIG 6:**
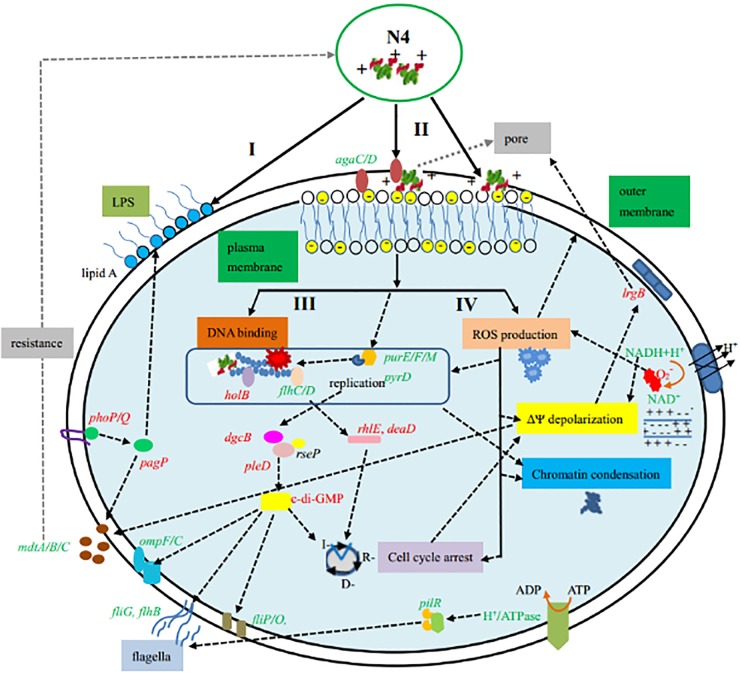
Mode of action for N4 in E. coli cells and differential correlation with expressed genes. Shown is the differential gene expression in E. coli during the whole N4 treatment process, which includes the following steps: I, binding to LPS; II, penetration of the outer membrane within 1 min and disintegration of the plasma membrane after 0.5 h of treatment; III, binding to DNA, intercalating into DNA base pairs, and inhibition of DNA and RNA synthesis within 15 min; and IV, inducing of apoptosis-like cell death within 0.5 h (including ROS production, depolarization of ΔΨ, chromatin condensation, and cell cycle arrest at the R and D phases). The genes shown in red had upregulated expression, and those shown in green had downregulated expression.

The I, R, and D phases of DNA in prokaryotic cells were equivalent to the G_1_, S, and M phases of eukaryotic cells (28). The cell cycle analysis showed that N4 caused the R- and D-phase cell cycle arrest of E. coli within 0.5 to 2 h (Fig. 2d and e), which indicates physiological changes induced by N4 after penetration of the cell membranes. It is possible that the increased DgcB activity, together with PleD activation, upshifts c-di-GMP to drive PopA-dependent CtrA degradation and R-phase entry (Fig. 6) (50). In addition, the cell cycle and flagellation are interdependent, and the *flhDC* gene is involved in coupling these processes. FlhD regulates flagella and the cell division rate (51). The regulation of the cell division rate by FlhD involves the acid response gene *cadA*, encoding lysine decarboxylase (51). In this study, the *ldcC* and *cadA* genes related to lysine degradation were upregulated 1.2- to 1.4-fold after the N4 treatment, which indicated that the membrane was disrupted (52).

Generation of ROS is a common mechanism causing bacterial death in most classes of bactericidal antibiotics (53). Kolodkin-Gal et al. reported that the ROS formation pathway triggered by antibiotics led to cell death in E. coli (53). Using DHR-123 staining, for the first time, we confirmed a significant increase in intracellular ROS generation in response to N4 treatment within 0.5 h (Fig. 3a). N4 might trigger disruption of the plasma membrane directly or indirectly by inducing apoptosis-like cell death via intracellular ROS accumulation (Fig. 6), which may cause lethal membrane depolarization (54).

ΔΨ, which plays a critical role in bacterial physiology, has been successfully applied to the viability assessment of a range of bacterial species (55). Determination of ΔΨ indicated that N4 induced depolarization of the bacterial cytoplasmic membrane within 1 h (Fig. 3b), which was consistent with the results for the magainin 2, pseudin-2, melittin, indolicidin, CP29, and CP11CN peptides against bacteria (33, 56, 57). N4 induced the transcription of *lrgB*, which is consistent with that of telavancin, daptomycin, carbonyl cyanide m-chlorophenylhydrazone (CCCP), and chitosan (58, 59), which indicated a loss of membrane integrity (59). LrgB is also potentially involved in pore formation and detoxification (58). However, in our study, downregulated NADH dehydrogenase may significantly contribute to the change in ΔΨ and reduced respiration (*ttrB*) may lead to a decline in energy consumption (Fig. 4c and 6) (54).

Apoptotic chromosomal DNA condensation is frequently an integral part of apoptosis in higher organisms (58). This study reported that apoptotic chromatin changes were exhibited in E. coli cells after incubation with 1× MIC N4 for 0.5 h (Fig. 3d) in accordance with earlier reports for arenicin-1, papiliocin, and psacotheasin against C. albicans (48, 52, 59). It is possible that the generation of ROS by N4 triggered the apoptosis-like process within 0.5 h, including cell cycle arrest, plasma membrane depolarization, and chromatin condensation (4) (Fig. 6).

It is worth noting that N4 induced multidrug resistance protein genes, such as *mdtA*, *mdtB*, and *mdtC* (Table S5), which is inconsistent with arenicin-3 (see Table S6 in the supplemental material) (14). The PhoP-PhoQ-activated *pagP* gene in this study was identified as important for inducible cationic AMP resistance and increased acylation of lipid A (Fig. 6). Increased acylation of lipid A is predicted to alter the fluidity of the outer membrane by increasing hydrophobic interactions between increased numbers of lipid A acyl tails (37). Although mutants of E. coli resistant to N4 were not detected in this study, the molecular basis for the above-mentioned resistance genes and other possible resistance mechanisms need further study.

Based on the obtained results, we propose a potential multiple-hit mechanism induced by N4 from marine resources: (i) binding to LPS, which increased acylation of lipid A; (ii) penetration of the outer membrane within 1 min, resulting in the disintegration of the plasma membrane after 0.5 h of treatment and the release of the cell contents; (iii) entering the cytoplasm within 5 min, where it became bound with DNA, changed conformation, and inhibited DNA and RNA synthesis within 15 min; and (iv) exhibition of some hallmarks of apoptosis-like cell death within 0.5 h via cell cycle arrest, ROS production, plasma membrane depolarization, and chromatin condensation (Fig. 6). A large number of genes are involved in the response to the destabilized membrane and DNA binding (Fig. 6). N4 promoted the survival ratio of bacterial peritonitis and endotoxemic mice, inhibited the release of cytokines, and protected the lungs from damage by LPS. It also proves that N4 has low cytotoxicity and no resistance, making it a promising candidate for development as a novel multitarget therapeutic agent against Gram-negative bacteria and endotoxemia.

## MATERIALS AND METHODS

### Structure determination of N4.

The secondary structure of N4 was determined and is described in detail in the supplemental material.

### Antimicrobial activity, cytotoxicity, and resistance of N4.

The MIC and minimal bactericidal concentration (MBC) values of N4 against bacterial strains and fungi and the MIC value of N4 treated with LPS against E. coli CVCC195 were determined using a broth microdilution technique as previously described (60).

To determine the effect of N4 on the viability of porcine intestinal epithelial cells ZYM-SIEC02 and mouse peritoneal macrophages RAW264.7 cells (1 × 10^4^ cells/ml), colorimetric MTT assays were performed in the Laboratory of Anshan Shan at Northeast Agricultural University (Harbin, China) and our laboratory, respectively, according to a previous method (61).

The resistance experiment for N4 was performed by sequential passaging. These methods are described in detail in the supplemental material.

### Binding affinities to LPS.

The MIC and probe displacement methods were used to determine the affinities of binding of the compounds to LPS as described in detail in the supplemental material.

### Interaction of N4 with the E. coli membrane. (i) Outer membrane permeabilization assays.

The outer membrane permeabilization activity of N4 was investigated by an NPN uptake assay. The hydrophobic antibiotic rifampin in association with N4 was used to detect the outer membrane permeability, which was tested using a synergistic growth inhibition assay as described in detail in the supplemental material.

### (ii) Flow cytometric analysis of plasma membrane permeability.

The E. coli cells at the mid-log phase (10^8^ CFU/ml) were collected by centrifugation at 5,500 × *g* for 5 min and resuspended in 0.01 M PBS (pH 7.4). The cells were incubated with 1× MIC N4 or FITC-labeled N4 at 37°C for 5 min, 0.5 h, and 2 h. After incubation, the cells were washed twice with PBS and resuspended in 450 μl of PBS. To determine the integrity of the cell membrane, 50 μl of 0.5 mg/ml PI was added to the cells (the cells were treated with FITC-labeled N4 without the addition of PI) and gently mixed. After incubation at room temperature for 20 min, the analysis was performed with a FACS Calibur flow cytometer (Becton Dickinson, San Jose, CA, USA).

### (iii) Measurement of the released DNA.

The amounts of DNA released from the E. coli cells treated with N4 were measured by optical density at 260 nm (OD_260_) and OD_280_ using a UV spectrophotometer (Amersham Pharmacia Biotech) as described in detail in the supplemental material.

### Interaction of N4 with E. coli DNA.

The genomic DNA was extracted from E. coli using a TIANamp Bacteria DNA kit (Tiangen). The interaction of N4 with E. coli DNA was conducted by the gel retardation experiments and CD spectra (62), respectively, as described in detail in the supplemental material.

### Effect of N4 on the macromolecular synthesis.

The effect of N4 on the rate of label incorporation into the major biosynthetic pathways of E. coli was measured to determine the specificity of action of N4. The E. coli CVCC195 cells at the mid-log phase (10^5^ CFU/ml) were incubated with 1× MIC N4 or antibiotics at 37°C for 15 min. The radioactive precursors of [^3^H]thymidine, [^3^H]uridine, [^3^H]leucine, and [^3^H]glucosamine (40 μCi/ml) were added into the cells to measure DNA, RNA, protein, and peptidoglycan, respectively, and the mixtures were incubated for 20 min at 37°C. Ice-cold 25% trichloroacetic acid (TCA) was added into the mixture, which was placed on ice for 30 min. After centrifugation, the pellets were washed twice with 25% TCA, dried, and counted with scintillation fluid using a MicroBeta 1450 scintillation counter (PerkinElmer Inc., Waltham, MA, USA).

### Markers of apoptosis-like cell death of E. coli cells induced by N4. (i) Cell cycle analysis by flow cytometry.

The DNA contents of the cells treated with N4 were quantified using a PI flow cytometric assay (48) as described in detail in the supplemental material. The data were analyzed using ModFit software.

### (ii) Reactive oxygen species accumulation.

The intracellular ROS production was measured using a fluorescent dye, DHR-123, which is oxidized to a fluorescent derivative, RH-123, so that an increase in the fluorescent signal reflects the ROS accumulation (48).

### (iii) Plasma membrane potential.

The plasma membrane depolarization was assessed by measuring the uptake of RH-123 fluorescent dye (31).

### (iv) Chromatin condensation.

The chromatin condensation was analyzed by staining with DAPI dyes and using a nucleic acid probe that displays a 20-fold-enhanced fluorescence upon DNA binding (31). These methods, mentioned above, are described in detail in the supplemental material.

### Transmission electron microscopy.

For TEM, the exponential-phase E. coli (1 × 10^8^ CFU/ml) cells were treated with 4× MIC N4 for 2 h at 37°C. The cells were fixed, dehydrated, and stained as described in detail in the supplemental material.

### RNA isolation, library preparation, and Illumina sequencing.

E. coli (10^8^ CFU/ml) cells were cultured in the presence of 1× MIC N4 or PBS for 0.5 h, 1 h, and 2 h. The total RNA extraction and RNA-sequencing library preparation were performed according to Illumina's protocols. RNA sequencing and data analysis were performed on the Illumina HiSeq 2000 platform at the Beijing Genome Institute (BGI) (Shenzhen, China).

### Mouse *in vivo* experiments. (i) The peritonitis model in mice.

Female ICR mice (6 weeks old; 10 groups each containing eight animals) were intraperitoneally injected with E. coli CVCC1515 (2.5 × 10^8^ CFU/ml, 1 ml). Mice were intraperitoneally injected with N4 or polymyxin B (PMB) (0.155, 0.31, 0.625, 1.25, or 2.5 mg/kg of body weight, 0.2 ml) at 0.5 and 8 h after inoculation of E. coli, respectively. Mice injected with only E. coli or saline served as positive or blank controls, respectively. The survival of the mice was recorded every 12 h and monitored for up to 7 days.

### (ii) The LPS-induced endotoxemia model in mice.

Specific pathogen-free C57BL/6 mice (6 to 8 weeks old) were purchased from Vital River Laboratories (VRL; Beijing, China). The mice were cared for in accordance with the institutional guidelines from the Animal Care and Use Committee of the Feed Research Institute, Chinese Academy of Agricultural Sciences (Beijing, China), and the experimental procedure was approved by the committee. The mice were intraperitoneally injected with LPS (10 and 30 mg/kg of body weight) from E. coli 0111:B4 followed by injection with N4 (2.5 and 5 mg/kg of body weight) or PMB (10 and 15 mg/kg of body weight) at 0.5 h and 8 h after inoculation, respectively. The mice received an intraperitoneal injection twice with PBS (0.1 ml), which served as a blank control. The survival of the mice was recorded every 2 h and monitored for up to 7 days.

Sera were collected from the mice sacrificed at 2 h and 8 h after injection with LPS. The levels of IL-6, IL-1β, and TNF-α in serum were determined at Jiaxuan Biotech. Co. Ltd. (Beijing, China), using an enzyme-linked immunosorbent assay (ELISA) kit and according to the manufacturer's protocol.

Lung specimens were dissected at 96 h after treatment, washed in PBS, and fixed in 4% paraformaldehyde at 4°C for 24 h. After rinsing with PBS and dehydrating with a series of ethanol solutions (75% to 95%), the tissues were infiltrated with xylene, embedded in paraffin wax, sectioned, and stained with hematoxylin and eosin. The samples were observed using a Nikon microscope.

### Statistical analysis.

All of the data were analyzed and performed using analysis of variance (ANOVA) models in SAS 9.2 (SAS Institute Inc., Cary, NC, USA). A *P* value of <0.05 was considered statistically significant.

## Supplementary Material

Supplemental material

## References

[1] Ababneh M, Harpe S, Oinonen M, Polk RE 2012 Trends in aminoglycoside use and gentamicin-resistant gram-negative clinical isolates in US academic medical centers: implications for antimicrobial stewardship. Infect Control Hosp Epidemiol 33:1–20. doi:10.1086/665724. 22561715

[2] Takeyama N, Yuki Y, Tokuhara D, Oroku K, Mejima M, Kurokawa S, Kuroda M, Kodama T, Nagai S, Ueda S, Kiyono H 2015 Oral rice-based vaccine induces passive and active immunity against enterotoxigenic *E. coli*-mediated diarrhea in pigs. Vaccine 33:5204–5211. doi:10.1016/j.vaccine.2015.07.074. 26254309

[3] van Langevelde P, Kwappenberg KM, Groeneveld PH, Mattie H, van Dissel JT 1998 Antibiotic-induced lipopolysaccharide (LPS) release from *Salmonella typhi*: delay between killing by ceftazidime and imipenem and release of LPS. Antimicrob Agents Chemother 42:739–743. 9559775 10.1128/aac.42.4.739 PMC105534

[4] Li P, Wohland T, Ho B, Ding JL 2004 Perturbation of lipopolysaccharide (LPS) micelles by Sushi 3 (S3) antimicrobial peptide. The importance of an intermolecular disulfide bond in S3 dimer for binding, disruption, and neutralization of LPS. J Biol Chem 279:50150–50156. 15328339 10.1074/jbc.M405606200

[5] Pulido D, Nogués MV, Boix E, Torrent M 2012 Lipopolysaccharide neutralization by antimicrobial peptides: a gambit in the innate host defense strategy. J Innate Immun 4:327–336. doi:10.1159/000336713. 22441679 PMC6741597

[6] Ponnappan N, Budagavi DP, Yadav BK, Chugh A 2015 Membrane-active peptides from marine organisms-antimicrobials, cell-penetrating peptides and peptide toxins: applications and prospects. Probiotics Antimicrob Proteins 7:75–89. doi:10.1007/s12602-014-9182-2. 25559972

[7] Hoegenhaug HHK, Mygind PH, Kruse T, Segura DR, Sandvang D, Neve S 12 2011 Antimicrobial peptide variants and polynucleotides encoding same. US patent 20110306750 A1.

[8] Andrä J, Jakovkin I, Grötzinger J, Hecht O, Krasnosdembskaya AD, Goldmann T, Gutsmann T, Leippe M 2008 Structure and mode of action of the antimicrobial peptide arenicin. Biochem J 410:113–122. doi:10.1042/BJ20071051. 17935487

[9] Ovchinnikova TV, Shenkarev ZO, Balandin SV, Nadezhdin KD, Paramonov AS, Kokryakov VN, Arseniev AS 2008 Molecular insight into mechanism of antimicrobial action of the beta-hairpin peptide arenicin: specific oligomerization in detergent micelles. Biopolymers 89:455–464. doi:10.1002/bip.20865. 17937399

[10] Choi H, Rangarajan N, Weisshaar JC 2016 Lights, camera, action! Antimicrobial peptide mechanisms imaged in space and time. Trends Microbiol 24:111–122. doi:10.1016/j.tim.2015.11.004. 26691950 PMC4733415

[11] Hutter B, Schaab C, Albrecht S, Borgmann M, Brunner NA, Freiberg C, Ziegelbauer K, Rock CO, Ivanov I, Loferer H 2004 Prediction of mechanisms of action of antibacterial compounds by gene expression profiling. Antimicrob Agents Chemother 48:2838–2844. doi:10.1128/AAC.48.8.2838-2844.2004. 15273089 PMC478524

[12] Hong RW, Shchepetov M, Weiser JN, Axelsen PH 2003 Transcriptional profile of the *Escherichia coli* response to the antimicrobial insect peptide cecropin A. Antimicrob Agents Chemother 47:1–6. doi:10.1128/AAC.47.1.1-6.2003. 12499161 PMC149021

[13] Nielsen AK, Sandvang D, Neve S, Kruse T, Kristensen H-H 2010 Transcriptional profiling indicates a dual mode-of-action of Arenicin-3, poster F1-2072. 50th Intersci Conf Antimicrob Agents Chemother, 12 to 15 September 2010. American Society for Microbiology, Washington, DC.

[14] Kozlowska J, Vermeer LS, Rogers GB, Rehnnuma N, Amos SB, Koller G, McArthur M, Bruce KD, Mason AJ 2014 Combined systems approaches reveal highly plastic responses to antimicrobial peptide challenge in *Escherichia coli*. PLoS Pathog 10:e1004104. doi:10.1371/journal.ppat.1004104. 24789011 PMC4006907

[15] Stavrakoudis A, Tsoulos IG, Shenkarev ZO, Ovchinnikova TV 2009 Molecular dynamics simulation of antimicrobial peptide arenicin-2: beta-hairpin stabilization by noncovalent interactions. Biopolymers 92:143–155. doi:10.1002/bip.21149. 19189382

[16] Wu EL, Fleming PJ, Yeom MS, Widmalm G, Klauda JB, Fleming KG, Im W 2014 *E. coli* outer membrane and interactions with OmpLA. Biophys J 106:2493–2502. doi:10.1016/j.bpj.2014.04.024. 24896129 PMC4052237

[17] Bhunia A, Domadia PN, Torres J, Hallock KJ, Ramamoorthy A, Bhattacharjya S 2010 NMR structure of Pardaxin, a pore-forming antimicrobial peptide, in lipopolysaccharide micelles. J Biol Chem 285:3883–3895. doi:10.1074/jbc.M109.065672. 19959835 PMC2823531

[18] Lohner K, Prenner EJ 1999 Differential scanning calorimetry and X-ray diffraction studies of the specificity of the interaction of antimicrobial peptides with membrane-mimetic systems. Biochim Biophys Acta 1462:141–156. doi:10.1016/S0005-2736(99)00204-7. 10590306

[19] Bouhdid S, Abrini J, Amensour M, Zhiri A, Espuny MJ, Manresa A 2010 Functional and ultrastructural changes in *Pseudomonas aeruginosa* and *Staphylococcus aureus* cells induced by *Cinnamomum verum* essential oil. J Appl Microbiol 109:1139–1149. doi:10.1111/j.1365-2672.2010.04740.x. 20456525

[20] Andrä J, Hammer MU, Grötzinger J, Jakovkin I, Lindner B, Vollmer E, Fedders H, Leippe M, Gutsmann T 2009 Significance of the cyclic structure and of arginine residues for the antibacterial activity of arenicin-1 and its interaction with phospholipid and lipopolysaccharide model membranes. Biol Chem 390:337–349. doi:10.1515/BC.2009.039. 19199831

[21] Kagan BL, Selsted ME, Ganz T, Lehrer RI 1990 Antimicrobial defensin peptides form voltage-dependent ion-permeable channels in planar lipid bilayer membranes. Proc Natl Acad Sci U S A 87:210–214. doi:10.1073/pnas.87.1.210. 1688654 PMC53231

[22] Wu M, Maier E, Benz R, Hancock RE 1999 Mechanism of interaction of different classes of cationic antimicrobial peptides with planar bilayers and with the cytoplasmic membrane of *Escherichia coli*. Biochemistry 38:7235–7242. doi:10.1021/bi9826299. 10353835

[23] Subbalakshmi C, Sitaram N 1998 Mechanism of antimicrobial action of indolicidin. FEMS Microbiol Lett 160:91–96. doi:10.1111/j.1574-6968.1998.tb12896.x. 9495018

[24] Tang YL, Shi YH, Zhao W, Hao G, Le GW 2009 Interaction of MDpep9, a novel antimicrobial peptide from Chinese traditional edible larvae of housefly, with *Escherichia coli* genomic DNA. Food Chem 115:867–872. doi:10.1016/j.foodchem.2008.12.102.

[25] Nagababu P, Shilpa M, Latha JN, Bhatnagar I, Srinivas PN, Kumar YP, Reddy KL, Satyanarayana S 2011 Synthesis, characterization, DNA binding properties, fluorescence studies and toxic activity of cobalt(III) and ruthenium(II) polypyridyl complexes. J Fluoresc 21:563–572. doi:10.1007/s10895-010-0743-9. 20931268

[26] Dehkordi MN, Bordbar AK, Mehrgardi MA, Mirkhani V 2011 Spectrophotometric study on the binding of two water soluble Schiff base complexes of Mn(III) with ct-DNA. J Fluoresc 21:1649–1658. doi:10.1007/s10895-011-0854-y. 21365249

[27] Higgins ML, Shockman GD 1971 Procaryotic cell division with respect to wall and membranes. CRC Crit Rev Microbiol 1:29–72. doi:10.3109/10408417109104477. 5004998

[28] Wu XZ, Chang WQ, Cheng AX, Sun LM, Lou HX 2010 Plagiochin E, an antifungal active macrocyclic bis (bibenzyl), induced apoptosis in *Candida albicans* through a metacaspase-dependent apoptotic pathway. Biochim Biophys Acta 1800:439–447. doi:10.1016/j.bbagen.2010.01.001. 20064588

[29] Nguyen LT, Haney EF, Vogel HJ 2011 The expanding scope of antimicrobial peptide structures and their modes of action. Trends Biotechnol 29:464–472. doi:10.1016/j.tibtech.2011.05.001. 21680034

[30] Choi H, Yang Z, Weisshaar JC 2015 Single-cell, real-time detection of oxidative stress induced in *Escherichia coli* by the antimicrobial peptide CM15. Proc Natl Acad Sci U S A 112:E303–E310. doi:10.1073/pnas.1417703112. 25561551 PMC4311848

[31] Wang X, Wang X, Teng D, Zhang Y, Mao R, Xi D, Wang J 2014 Candidacidal mechanism of the arenicin-3-derived peptide NZ17074 from *Arenicola marina*. Appl Microbiol Biotechnol 98:7387–7398. doi:10.1007/s00253-014-5784-6. 24816779

[32] Hakansson AP, Roche-Hakansson H, Mossberg AK, Svanborg C 2011 Apoptosis-like death in bacteria induced by HAMLET, a human milk lipid-protein complex. PLoS One 6:e17717. doi:10.1371/journal.pone.0017717. 21423701 PMC3053380

[33] Lee W, Lee DG 2014 Magainin 2 induces bacterial cell death showing apoptotic properties. Curr Microbiol 69:794–801. doi:10.1007/s00284-014-0657-x. 25023640

[34] Anderson RC, Hancock RE, Yu PL 2004 Antimicrobial activity and bacterial-membrane interaction of ovine-derived cathelicidins. Antimicrob Agents Chemother 48:673–676. doi:10.1128/AAC.48.2.673-676.2004. 14742236 PMC321555

[35] Pinheiro da Silva F, Medeiros MC, Dos Santos ÂB, Ferreira MA, Garippo AL, Chammas R, Caldini E, Velasco IT, Possolo de Souza H, Machado MC 2013 Neutrophils LL-37 migrate to the nucleus during overwhelming infection. Tissue Cell 45:318–320. doi:10.1016/j.tice.2013.04.003. 23742816

[36] Sperandio V, Torres AG, Kaper JB 2002 Quorum sensing *Escherichia coli* regulators B and C (QseBC): a novel two-component regulatory system involved in the regulation of flagella and motility by quorum sensing in *E. coli*. Mol Microbiol 43:809–821. doi:10.1046/j.1365-2958.2002.02803.x. 11929534

[37] Bren A, Eisenbach M 2000 How signals are heard during bacterial chemotaxis: protein-protein interactions in sensory signal propagation. J Bacteriol 182:6865–6873. doi:10.1128/JB.182.24.6865-6873.2000. 11092844 PMC94809

[38] Burton GJ, Hecht GB, Newton A 1997 Roles of the histidine protein kinase pleC in *Caulobacter crescentus* motility and chemotaxis. J Bacteriol 179:5849–5853. 9294444 10.1128/jb.179.18.5849-5853.1997 PMC179476

[39] Loutet SA, Di Lorenzo F, Clarke C, Molinaro A, Valvano MA 2011 Transcriptional responses of *Burkholderia cenocepacia* to polymyxin B in isogenic strains with diverse polymyxin B resistance phenotypes. BMC Genomics 12:472. doi:10.1186/1471-2164-12-472. 21955326 PMC3190405

[40] Postma PW, Lengeler JW, Jacobson GR 1993 Phosphoenolpyruvate:carbohydrate phosphotransferase systems of bacteria. Microbiol Rev 57:543–594. 8246840 10.1128/mr.57.3.543-594.1993 PMC372926

[41] Nagakubo S, Nishino K, Hirata T, Yamaguchi A 2002 The putative response regulator BaeR stimulates multidrug resistance of *Escherichia coli* via a novel multidrug exporter system, MdtABC. J Bacteriol 184:4161–4167. doi:10.1128/JB.184.15.4161-4167.2002. 12107133 PMC135206

[42] Wang X, Wang H, Xie J 2011 Genes and regulatory networks involved in persistence of *Mycobacterium tuberculosis*. Sci China Life Sci 54:300–310. doi:10.1007/s11427-011-4134-5. 21267668

[43] Guo L, Lim KB, Poduje CM, Daniel M, Gunn JS, Hackett M, Miller SI 1998 Lipid A acylation and bacterial resistance against vertebrate antimicrobial peptides. Cell 95:189–198. doi:10.1016/S0092-8674(00)81750-X. 9790526

[44] Kim KS, Yang HJ, Choi EK, Park YJ, Cho DH, Ahn KS 2011 The multi-target antibiotic efficacy of *Angelica dahurica* Bentham et Hooker extract exposed to the *Escherichia coli* O157:H7. BioChip J 5:333–342. doi:10.1007/s13206-011-5407-6.

[45] Blondelle SE, Lohner K, Aguilar M 1999 Lipid-induced conformation and lipid-binding properties of cytolytic and antimicrobial peptides: determination and biological specificity. Biochim Biophys Acta 1462:89–108. doi:10.1016/S0005-2736(99)00202-3. 10590304

[46] Pinheiro da Silva F, Gallo RL, Nizet V 2009 Differing effects of exogenous or endogenous cathelicidin on macrophage toll-like receptor signaling. Immunol Cell Biol 87:496–500. doi:10.1038/icb.2009.19. 19350049 PMC2763337

[47] Hu L, Sun C, Wang S, Su F, Zhang S 2013 Lipopolysaccharide neutralization by a novel peptide derived from phosvitin. Int J Biochem Cell Biol 45:2622–2631. doi:10.1016/j.biocel.2013.09.002. 24028820

[48] Chang DK, Cheng SF, Chien WJ 1997 The amino-terminal fusion domain peptide of human immunodeficiency virus type 1 gp41 inserts into the sodium dodecyl sulfate micelle primarily as a helix with a conserved glycine at the micelle-water interface. J Virol 71:6593–6602. 9261381 10.1128/jvi.71.9.6593-6602.1997 PMC191937

[49] Matsuzaki K, Yoneyama S, Miyajima K 1997 Pore formation and translocation of melittin. Biophys J 73:831–838. doi:10.1016/S0006-3495(97)78115-3. 9251799 PMC1180979

[50] Abel S, Chien P, Wassmann P, Schirmer T, Kaever V, Laub MT, Baker TA, Jenal U 2011 Regulatory cohesion of cell cycle and cell differentiation through interlinked phosphorylation and second messenger networks. Mol Cell 43:550–560. doi:10.1016/j.molcel.2011.07.018. 21855795 PMC3298681

[51] Chilcott GS, Hughes KT 2000 Coupling of flagellar gene expression to flagellar assembly in *Salmonella enterica* serovar typhimurium and *Escherichia coli*. Microbiol Mol Biol Rev 64:694–708. doi:10.1128/MMBR.64.4.694-708.2000. 11104815 PMC99010

[52] Andrä J, Goldmann T, Ernst CM, Peschel A, Gutsmann T 2011 Multiple peptide resistance factor (MprF)-mediated resistance of *Staphylococcus aureus* against antimicrobial peptides coincides with a modulated peptide interaction with artificial membranes comprising lysyl-phosphatidylglycerol. J Biol Chem 286:18692–18700. doi:10.1074/jbc.M111.226886. 21474443 PMC3099686

[53] Kolodkin-Gal I, Sat B, Keshet A, Engelberg-Kulka H 2008 Communication factor EDF and the toxin-antitoxin mazEF determine the mode of action of antibiotics. PLoS Biol 6(12):e319. doi:10.1371/journal.pbio.0060319. 19090622 PMC2602726

[54] Liu W, Dong SL, Xu F, Wang XQ, Withers TR, Yu HD, Wang X 2013 Effect of intracellular expression of antimicrobial peptide LL-37 on growth of *Escherichia coli* strain TOP10 under aerobic and anaerobic conditions. Antimicrob Agents Chemother 57:4707–4716. doi:10.1128/AAC.00825-13. 23856776 PMC3811468

[55] Porter J, Diaper J, Edwards C, Pickup R 1995 Direct measurements of natural planktonic bacterial community viability by flow cytometry. Appl Environ Microbiol 61:2783–2786. 16535084 10.1128/aem.61.7.2783-2786.1995 PMC1388502

[56] Friedrich CL, Moyles D, Beveridge TJ, Hancock RE 2000 Antibacterial action of structurally diverse cationic peptides on Gram-positive bacteria. Antimicrob Agents Chemother 44:2086–2092. doi:10.1128/AAC.44.8.2086-2092.2000. 10898680 PMC90018

[57] Park SC, Kim JY, Jeong C, Yoo S, Hahm KS, Park Y 2011 A plausible mode of action of pseudin-2, an antimicrobial peptide from *Pseudis paradoxa*. Biochim Biophys Acta 1808:171–182. doi:10.1016/j.bbamem.2010.08.023. 20826126

[58] Song Y, Lunde CS, Benton BM, Wilkinson BJ 2012 Further insights into the mode of action of the lipoglycopeptide telavancin through global gene expression studies. Antimicrob Agents Chemother 56:3157–3164. doi:10.1128/AAC.05403-11. 22411615 PMC3370745

[59] Hwang B, Hwang JS, Lee J, Kim JK, Kim SR, Kim Y, Lee DG 2011 Induction of yeast apoptosis by an antimicrobial peptide, Papiliocin. Biochem Biophys Res Commun 408:89–93. doi:10.1016/j.bbrc.2011.03.125. 21458420

[60] Zhang Y, Teng D, Wang X, Mao R, Cao X, Hu X, Zong L, Wang J 2015 In vitro and in vivo characterization of a new recombinant antimicrobial peptide, MP1102, against methicillin-resistant *Staphylococcus aureus*. Appl Microbiol Biotechnol 99:6255–6266. doi:10.1007/s00253-015-6394-7. 25620367

[61] Jiao J, Mao RY, Wang XM, Zhang Y, Teng D, Feng XJ, Wang JH 2015 GAP-initiated constitutive expression of a novel plectasin-derived peptide MP1106 by *Pichia pastoris* and its activity against *Streptococcus suis*. Process Biochem 50:253–261. doi:10.1016/j.procbio.2014.12.019.

[62] Park CB, Kim HS, Kim SC 1998 Mechanism of action of the antimicrobial peptide buforin II: buforin II kills microorganisms by penetrating the cell membrane and inhibiting cellular functions. Biochem Biophys Res Commun 244:253–257. doi:10.1006/bbrc.1998.8159. 9514864

