# Supplementary material for: Combined Systems Approaches Reveal a Multistage Mode of Action of a Marine Antimicrobial Peptide against Pathogenic Escherichia coli and Its Protective Effect against Bacterial Peritonitis and Endotoxemia
Source: Antimicrob Agents Chemother. 2016 Dec 27;61(1):e01056-16. doi: 10.1128/AAC.01056-16 (PMC5192121; doi:10.1128/AAC.01056-16)
Supplement: Supplemental material [file supp_61_1_e01056-16__index.html]

Combined Systems Approaches Reveal a Multistage Mode of Action of a Marine Antimicrobial Peptide against Pathogenic Escherichia coli and Its Protective Effect against Bacterial Peritonitis and Endotoxemia — Supplemental material 

# Combined Systems Approaches Reveal a Multistage Mode of Action of a Marine Antimicrobial Peptide against Pathogenic Escherichia coli and Its Protective Effect against Bacterial Peritonitis and Endotoxemia

## Supplemental material

- Supplemental file 1 -

  Supplemental methods, Figures S1 to S3, and Tables S1 to S6

  PDF, 628K
