# Supplementary material for: Combined Systems Approaches Reveal a Multistage Mode of Action of a Marine Antimicrobial Peptide against Pathogenic Escherichia coli and Its Protective Effect against Bacterial Peritonitis and Endotoxemia
Source: Antimicrob Agents Chemother. 2016 Dec 27;61(1):e01056-16. doi: 10.1128/AAC.01056-16 (PMC5192121; doi:10.1128/AAC.01056-16)
Supplement: Supplemental material [file AAC.01056-16_zac001175805s1.pdf]

Supplemental Material

Combined systems approaches reveal a multistage mode of action of a marine antimicrobial peptide against pathogenic *Escherichia coli* and its protective effect against endotoxemia

Xiumin Wang[1, 2], Da Teng[1, 2], Ruoyu Mao[1, 2], Na Yang[1, 2], Ya Hao[1, 2], Jianhua Wang[1, 2*]

[1] Key Laboratory of Feed Biotechnology, Ministry of Agriculture, Beijing 100081, China

[2] Gene Engineering Laboratory, Feed Research Institute, Chinese Academy of Agricultural Sciences, Beijing 100081, China

* Corresponding author

Prof., Ph.D., and PI. Jianhua Wang

Gene Engineering Laboratory, Feed Research Institute, Chinese Academy of Agricultural Sciences, 12 Zhongguancun Nandajie St., Haidian District, Beijing 100081, P. R. China

E-mail address: wangjianhua@caas.cn; 2681298635@qq.com

Phone: 0086-10-82106081, 0086-10-82106079; Fax: 0086-10-82106079

Supplementary 1: Materials and Methods

Materials. The test strains of bacteria shown in Table 1 were purchased from the Institute of Microbiology, the Chinese Academy of Sciences and the China Center of Industrial Culture Collection. *Candida albicans* (CMCC2.2411) and *Saccharomyces cerevisiae* CMCC2.1546 were purchased from the China General Microbiological Culture Collection. The solid-phase synthesis of N4 and FITC-labeled N4 (> 90% purity) was performed by China Peptides Co., Ltd. (Shanghai, China). NPN, PI, DHR-123, RH-123 and DAPI were purchased from Sigma Chemical Co. (Beijing, China). The genome DNA extraction kit was purchased from Tiangen Biotech (Beijing) Co., Ltd. Porcine intestinal epithelial cell line ZYM-SIEC02 from the China Center for Type Culture Collection (CCTCC) with the CCTCC accession number of C201001 were stored in the Laboratory of Prof. Anshan Shan in the Institute of Animal Nutrition, Northeast Agricultural University (Harbin, China). The other reagents were of analytical grade. The *E. coli* CVCC 195 strain was used in all experiments of action of N4.

Structure determination of N4. The secondary structure of N4 was estimated with a Pistar π-180 circular dichroism (CD) spectrometer (Applied Photophysics Ltd.). The peptide was adjusted to 33 ng/μl in a 0.01 M sodium phosphate buffer (PBS) (pH 7.2) and measured in an aqueous or 10 mM sodium dodecyl sulfate (SDS) solution. The CD data were obtained from an average of four scans with a step size of 1 nm and a bandwidth of 3 nm. The spectra were recorded from 190 nm to 260 nm at a scanning rate of 10 nm/min with a constant time of 2 s (S1).

Antimicrobial activity, cytotoxicity and resistance of N4. The antimicrobial activities of N4 against bacterial strains and fungi were determined using an improved broth microdilution technique, as previously described (S2). The bacterial cells at the mid-log phase (10^8 CFU/ml) were diluted to 10^5 CFU/ml by fresh Mueller-Hinton (MH) broth. Two-fold serially diluted (0.625–640 μg/ml) aliquots of

N4 (10 μl/well) were added to sterile 96-well polypropylene microtiter plates, followed by the addition of 90 μl/well of above mid-log phase bacteria (10^5 CFU/ml). After incubation for 16–18 h at 37 ℃, the minimal inhibitory concentration (MIC) and minimal bactericidal concentration (MBC) were determined. The experiments were performed in triplicate.

To determine the effect of N4 on the viability of porcine intestinal epithelial cells ZYM-SIEC02 and mouse peritoneal macrophages RAW264.7 cells (1×10^4 cells/ml), the colorimetric MTT assay was performed in the Laboratory of Prof. Anshan Shan in Northeast Agricultural University (Harbin, China) and our laboratory, respectively according to the previous method (S3). Porcine intestinal epithelial cells (5×10^3 cells/well) were added into 96-well microtiter plates, and incubated in a humidified 5% (v/v) CO_2/air environment at 37 ℃ for 24 h. A 2-fold series of the N4 solutions ranging from 1 to 128 μg/ml were added into the plates and incubated for 48 h, and each concentration was evaluated in three separate wells. The untreated cells were used as controls. The MTT solution was added into plates (10 μl/well), incubated for 4 h, and then removed from plates. After addition of dimethyl sulfoxide (DMSO) (150 μl/well), the absorbance was measured at 570 nm with a spectrophotometer. The degree of inhibition of cell proliferation was calculated using the following formula: Growth inhibition (%) = $(Abs_{ODcontrol} - Abs_{ODtreated}) / Abs_{ODcontrol} \times 100$.

The exponential phase *E. coli* cells (10^6 CFU/ml) were treated with 0.25×, 0.5×, 1×, 2×, 4× and 8× MIC N4s, respectively. At 24 h intervals, cultures from the second highest concentrations (OD ≥ 0.5) were diluted 1:100 into fresh MHB media containing 0.25×, 0.5×, 1×, 2×, 4× and 8× MIC of N4. This serial passaging was repeated daily for 15 days. Any cultures that grew at higher than the MIC levels were passaged on peptide free MHA plates and the MIC was then determined by broth microdilution (S4). The resistance experiment of N4 was repeated for three times.

Binding affinities of N4 to lipopolysaccharides (LPS). The ability of N4 to neutralize LPS from *E. coli* CVCC195 *in vitro* was assayed using a modified broth microdilution technique as previously described (S5). N4 and LPS (w/w, 1: 4) mixture was incubated for 1 h at 37 ℃. Then, the MIC of N4 treated with LPS against *E. coli* CVCC195 was measured.

The BODIPY-TR-cadaverine (BC) displacement assay was used to determine the affinities of binding of compounds to LPS (S5). 50 μg/ml LPS from *E. coli* 0111:B4 (Sigma) and 10 μM BC were mixed in 5 mM HEPES buffer (pH 7.0). A series of peptide solutions were added into the mixture and incubated for 4 h at 37 ℃. The mixture was added into a 96-well black plate. Fluorescence was measured using a a Tecan Infinite M200 PRO plate reader at an excitation wavelength of 580 nm and an emission wavelength of 620 nm. Polymyxin B was used as the positive control.

Interaction of N4 with *E. coli* membrane.

Outer membrane permeabilization assays. The outer membrane permeabilization activity of N4 was investigated by an N-phenyl-1-naphthylamine (NPN) uptake assay, as described by Falla (S6). Briefly, 200 μl of an overnight culture of *E. coli* was added to 10 ml of fresh MHB and grown to an optical density at 600 nm of 0.4–0.6. The cells were washed with buffer (5 mM Sodium HEPES, 5 mM glucose, pH 7.2) and resuspended in the same buffer (OD_{600} = 0.5). For the real-time fluorescence measurements, 980 μl aliquots of cell suspension were prepared in tubes, and 10 μl of 1 mM NPN solution in acetone was added followed by 10 μl of N4 or an ampicillin aqueous solution. Subsequently, the above mixture was quickly pipetted into a black 96-well plate (300 μl/well). The fluorescence of NPN was monitored for 10 min on a Tecan Infinite M200 PRO plate reader with excitation at 345 nm and emission at 400 nm. The untreated cells were used as a blank control.

Measurement of the released DNA. The *E. coli* cells in the exponential phase (10^6 CFU/ml) were

treated with 1×, 2× and 4× MIC N4 at 37 ℃ for 1 h. The cells were filtered through 0.22-μm pore size filter and diluted 10 fold. Cells treated with 0.1 M PBS and 0.1% Triton X-100 were used as the negative and positive controls, respectively. The amounts of DNA released from the *E. coli* cells were measured by OD_{260} and OD_{280} using an ultraviolet spectrophotometer (Amersham Pharmacia Biotech).

Interaction of N4 with *E. coli* DNA. The genomic DNA was extracted from *E. coli* using a TIANamp Bacteria DNA kit (Tiangen). The gel retardation experiments were performed by mixing 0.5 μg of the genomic DNA with different concentrations of N4 (0, 0.25, 0.5, 1.0, 2.5 and 5 μg) in 20 μl of binding buffer (5% glycerol, 10 mM Tris-HCl pH 8.0, 1 mM EDTA, 1 mM dithiothreitol (DTT), 20 mM KCl, 50 μg/ml bovine serum albumin (BSA)). The reaction mixtures were incubated for 10 min at room temperature and then analyzed by 1% agarose gel electrophoresis (S7).

To examine whether N4 binding imparts conformational changes in *E. coli* genomic DNA, the CD spectra from 220 to 320 nm were recorded on a Pistar π-180 CD spectrometer using 1.0-mm path length quartz cell. The peptide to DNA mass ratios were 0, 1.25 and 5. The samples were run at 25 ℃, and the data are the average of 10 scans with an integration time of 20 s.

Apoptosis of *E. coli* cells induced by N4.

Cell cycle analysis by flow cytometry. The DNA contents of the cells were quantified by a PI flow cytometric assay (S7). The *E. coli* cells (10^8 CFU/ml) exposed to 1× MIC N4 for 0.5 h and 2 h were harvested by centrifugation at 5500× *g* and washed twice with 0.01 M PBS (pH 7.4) that was filtered through a 0.22-μm filter. Then, the cells were resuspended in 0.5 ml of PBS and fixed in 1.0 ml of cold 75% ethanol at 4 ℃ overnight. The cells were centrifuged, resuspended in 450 μl of PBS (containing 100 μg/ml of RNase A), and incubated at 37 ℃ for an additional 0.5 h. Then, 50 μl of PI solution (500 μg/ml) was added to the mixture and stained for 0.5 h in the dark. The DNA content and cell cycle

phase distribution were analyzed with a flow cytometer, and 30,000 cells were scored for each sample. The data were analyzed using ModFit software.

Intracellular reactive oxygen species (ROS) accumulation. The intracellular ROS production was measured using a fluorescent dye dihydrorhodamine-123 (DHR-123), which is oxidized to a fluorescent derivative, rhodamine-123 (RH-123), in such a manner that an increase in the fluorescent signal reflects the ROS accumulation (S7). The *E. coli* cells in the exponential phase (10^8 CFU/ml) were treated with 1× MIC N4 or 2.5 mM H_2O_2 for 0.5 h at 37℃. After incubation, the cells were washed twice with 0.01 M PBS (pH 7.4) before staining with 5 μg/ml of DHR-123. The changes in the fluorescent intensity of the cells were analyzed on a flow cytometer.

Plasma membrane potential ($\Delta\Psi$). The plasma membrane depolarization was assessed by measuring the uptake of RH-123 fluorescent dye, as described (S7). The log-phase cells (10^8 CFU/ml) were treated with or without 1× MIC N4 for 1 h. The cells were washed twice with PBS, resuspended in PBS before staining with 10 μg/ml RH-123 for 0.5 h at 37℃ in the dark, and analyzed using a flow cytometer.

Chromatin condensation. The chromatin condensation was analyzed by staining with a 4', 6-diamidino-2-phenylindole (DAPI) dye, a nucleic acid probe that displays a 20-fold enhanced fluorescence upon DNA binding (S7). The *E. coli* cells (10^8 CFU/ml) were incubated with 1× MIC N4 for 0.5 h at 37℃. The cells were washed twice with 10 mM of NaPB (pH 7.4) and incubated with DAPI (1 μg/ml) for 10 min. The cells were then harvested and examined by CLSM. Using this method, the chromatin DNA exhibits bright light blue fluorescence, whereas the other cytoplasmic portions of the cells and the background are black.

Transmission electron microscopy (TEM). The exponential phase *E. coli* (1×10^8 CFU/ml) cells

were treated with 4× MIC N4 for 2 h at 37℃. After centrifugation at 5500× *g* for 5 min, the cells were washed three times with PBS and fixed with 2.5% glutaraldehyde at 4℃ overnight. The cells were dehydrated in a graded series of ethanol, air-dried, mounted and sputter coated with carbon. These cells were post-fixed in 1% buffered osmium tetroxide for 1 h, stained with 1% uranyl acetate, subsequently dehydrated with a graded ethanol series, and embedded in Spur resin (S8). Thin sections were prepared on Formvar copper grids and stained with 2% uranyl acetate and then with lead citrate. The microscopy was performed with TEM (JEM-1400, JEDL, Tokyo, Japan).

Supplementary 2: Figures and Tables

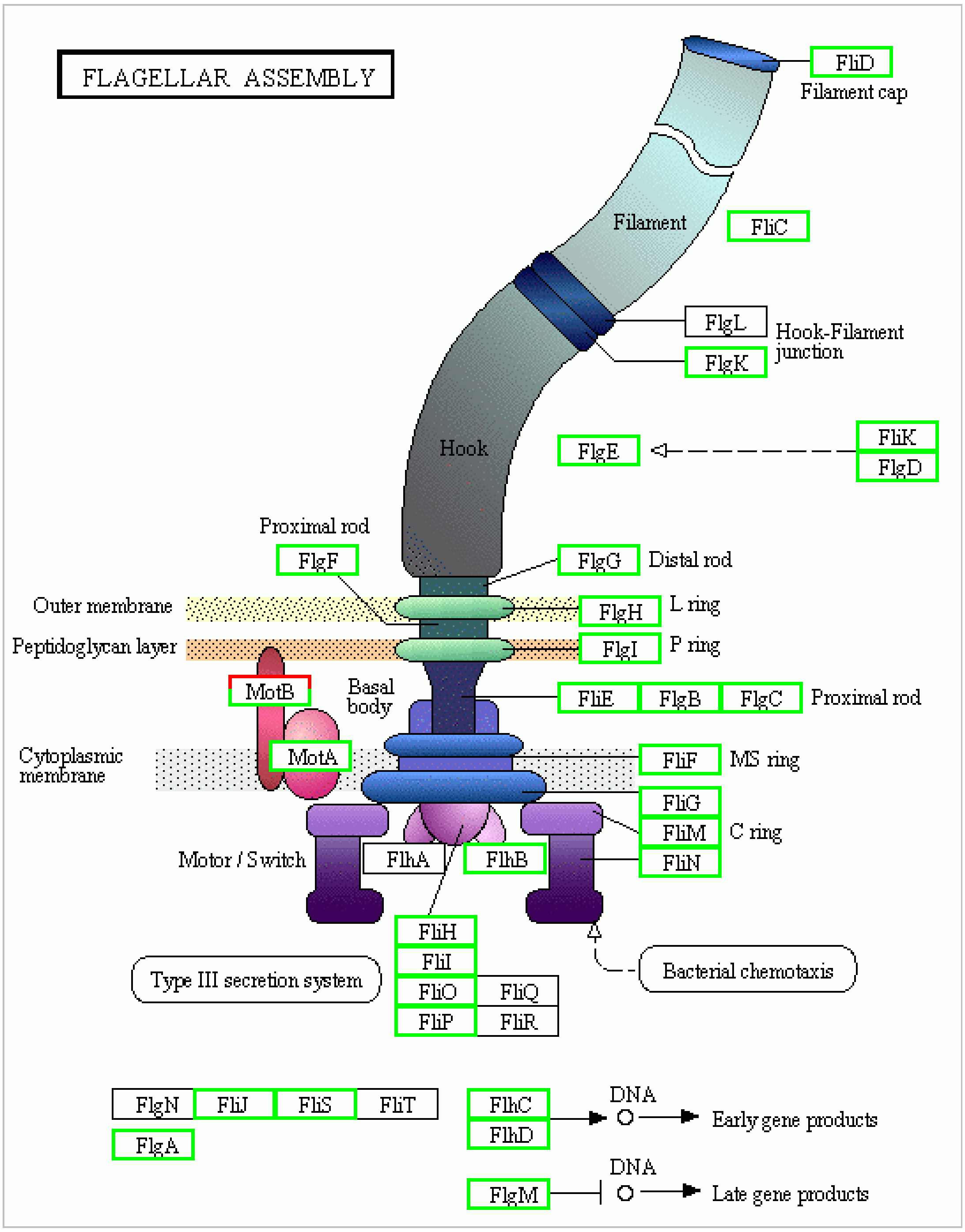

Supplementary FIG 1

Supplementary FIG 1 KEGG analysis of flagellar assembly in *E. coli* treated with N4 for 1 h. Red boxes indicate up-regulated genes, and green boxes indicate significantly down-regulated genes.

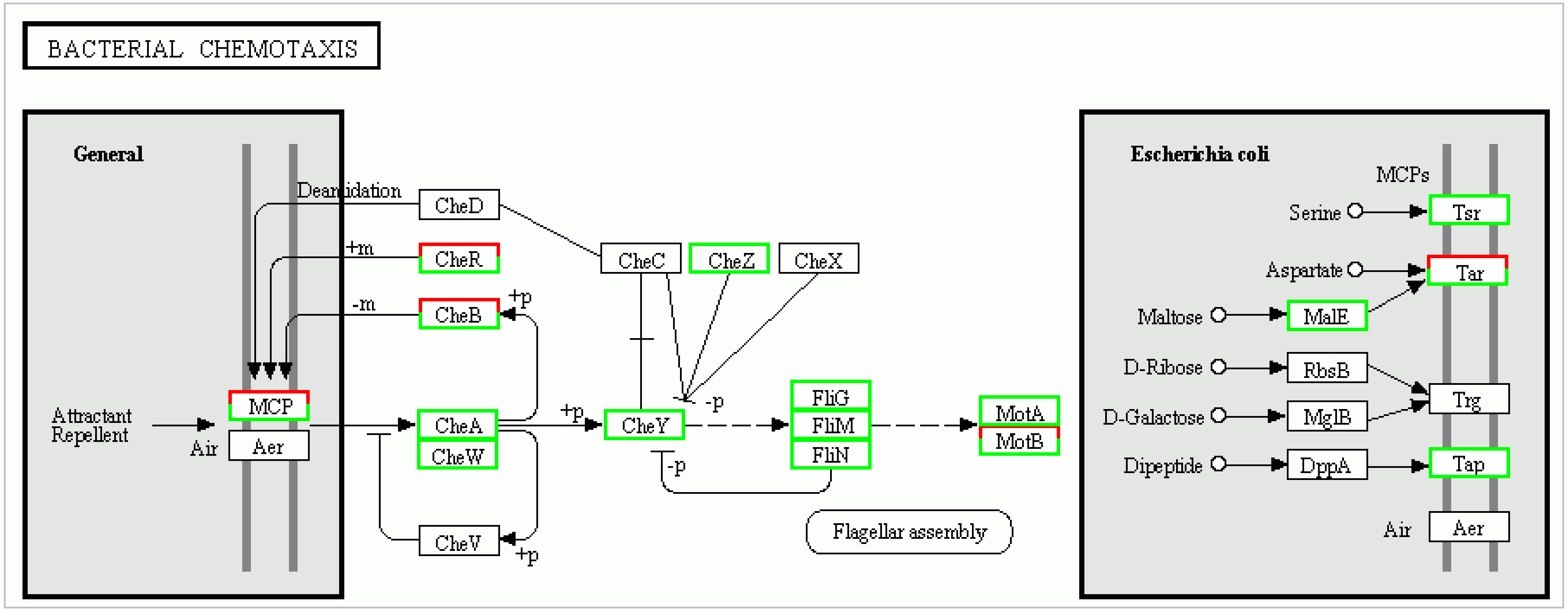

Supplementary FIG 2

Supplementary FIG 2 KEGG analysis of bacterial chemotaxis in *E. coli* treated with N4 for 1 h. Red boxes indicate up-regulated genes, and green boxes indicate significantly down-regulated genes.

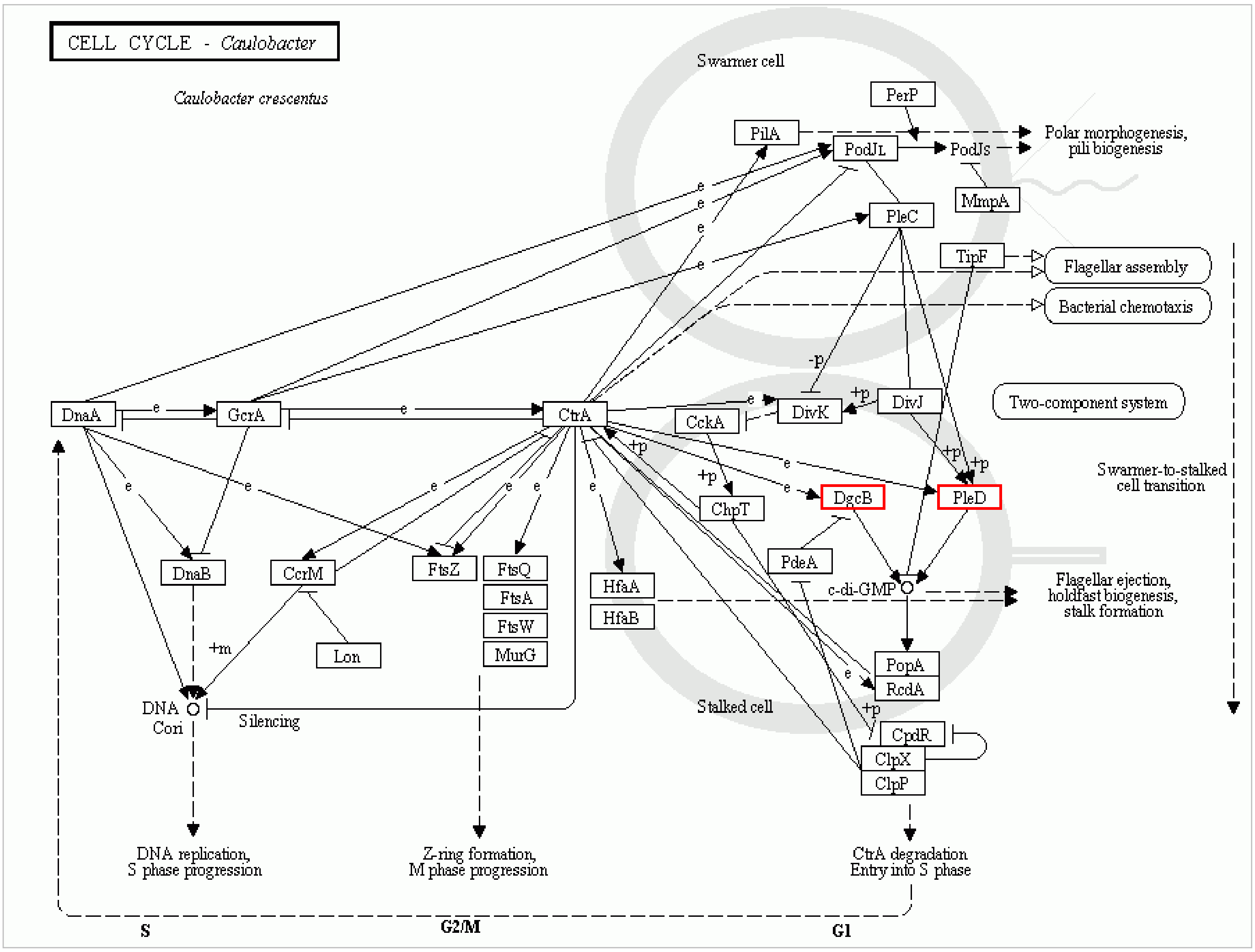

Supplementary FIG 3

Supplementary FIG 3 KEGG analysis of the cell cycle in *E. coli* treated with N4 for 1 h. Red boxes indicate up-regulated genes.

Supplementary TABLE 1 Cytotoxicity of N4 against mouse peritoneal macrophages RAW264.7 cells.

Concentration of N4 (μg/ml)	Inhibition rate (%) (Mean ± RSD)
1	1.1 ± 4.0
2	4.3 ± 0.2
4	7.7 ± 2.3
8	9.2 ± 1.4
16	22.7 ± 0.2
32	12.1 ± 0.7
64	43.0 ± 0.02
128	48.0 ± 0.2
256	48.2 ± 0.2

Supplementary TABLE 2 Description of significantly differential expressed membrane-associated genes in *E. coli* treated with N4 for 0.5 h, 1 h, and 2 h.

Gene	Full name	Gene	Full name
Outer membrane-associated genes			
ompF	porin protein OmpF	*lolA*	outer membrane lipocarrier protein LolA
amsH	amylovoran export OMP AmsH	*crcA*	palmitoyltransferase for lipid A CrcA
yiaD	inner membrane lipoprotein YiaD	*U7*	glucose dehydrogenase
flimC	chaperone protein FlimC	*modA*	molybdate ABC transporter, periplasmicmolybdate-binding protein ModA
ydcS	ABC transporter, periplasmic substrate-binding protein YdcB	*U8*	zinc-binding lipoprotein
U2	dipeptide transport protein	*lamB*	maltose-inducible porin protein LamB
artI	arginine ABC transporter periplasmic substrate-binding protein ArtI	*flgH*	flagellar L-ring family protein FlgH
omiA	OmiA family protein	*flgI*	flagellar P-ring family protein FlgI
potF	putrescine-binding periplasmic protein PotF	*ssuA*	alkanesulfonate transporter substrate-binding subunit SsuA
U3	fimbrial usher protein	*U9*	nucleoside-specific channel-forming protein
fhuA	ferrichrome outer membrane transporter FhuA	*ompC*	OMP OmpC
U4	lysine-arginine-ornithine-binding periplasmic protein	*nlpC*	lipoprotein NlpC
U5	Leu/Ile/Val/Thr-binding protein	*motB*	chemotaxis protein MotB
yaeT	OMP assembly factor YaeT	*U10*	class B acid phosphatase
gltI	glutamate/aspartate periplasmic-binding protein GltI	*ymcA*	outer membrane lipoprotein YmcA
glnH	glutamate ABC transporter periplasmic protein GlnH	*gfcE*	putative polysaccharide export protein GfcE
yncD	TonB-dependent receptor YncD	*U11*	outer membrane porin protein
U6	transglycosylase	*ompW*	OMP OmpW
ompX	OMP OmpX	*evgS*	sensor protein EvgS
yliF	diguanylatecyclaseYliF	*osmB*	osmotically-inducible lipoprotein B OsmB
ybaY	outer membrane lipoprotein YbaY	*htrA*	serine endoproteaseHtrA
aapJ	general L-amino acid-binding periplasmic protein AapJ		
Inner membrane-associated genes			
yiaD	IM lipoproteinYiaD	*yfdC*	IMP YfdC
yccA	IMP YccA	*yigG*	IMP YigG
yeeE	IMP YeeE	*ydhC*	IM transport protein YdhC

yehY	IM ABC trasporterpermeaseYehY	*ynjI*	IMP YnjI
yabI	IMP YabI	*ygjE*	IMP YgjE
yiaH	IMP YiaH	*yedE*	IMP YedE
yhhQ	IMP yhhQ	*nagE*	N-acetylglucosamine-specific EIICBA component protein NagE
ybhS	IM transporter permeaseYbhS	*yicO*	IMP YicO
ygbE	IMP YgbE	*gfcA*	threnonine-rich IMP GfcA
ygcS	IM metabolite transport protein YgcS	*vicO*	IMP VicO
creD	IMP CreD		

Supplementary TABLE 3 Description of significantly differential expressed flagellum-associated genes in *E. coli* treated with N4 for 0.5 h, 1 h, and 2 h.

Gene	Full name	Gene	Full name
fliC	flagellarproteinFliC	*flgI*	flagellar P-ring family protein FlgI
cheY	chemotaxis protein CheY	*fliJ*	flagellar export protein FliJ
cheZ	chemotaxis protein CheZ	*flgG*	flagellar basal-body rod protein FlgG
flgK	fagellar hook-associated protein FlgK	*fliK*	flagellar hook-length control protein FliK
flgL	fagellar hook-associated protein FlgL	*fliE*	flagellar hook-length control protein FliE
motA	chemotaxis protein MotA	*flgJ*	flagellar rod assembly protein FlgJ
motB	chemotaxis protein MotB	*flgD*	flagellar rod assembly protein FlgD
U1	flagellar cap protein	*fliH*	flagellar assembly protein FliH
fliS	flagellarproteinFliS	*flgE*	flagellar hook protein FlgE
fliR	flagellar biosynthetic protein FliR	*fliN*	flagellar motor switch protein FliN
flgB	flagellar basal-body rod protein FlgB	*fliF*	flagellar M-ring protein FliF
flgC	flagellar basal-body rod protein FlgC	*fliI*	flagellar protein export ATPase FliI
flgA	flagellar basal-bodyP-ring fromation protein FlgA	*fliM*	flagellar motor switch protein FliM
fliP	flagellar biosynthetic protein FliP	*fliL*	flagellar protein FliL
flgH	flagellar L-ring family protein FlgH	*fliG*	flagellar motor switch protein FliG

Supplementary TABLE 4 Description of significantly differential expressed DNA, RNA-associated genes in *E. coli* treated with N4 for 0.5 h, 1 h, and 2 h.

Gene	Full name	Gene	Full name
DNA-associated genes			
flhC	flagellar transcriptional activator FlhC	*rpoE*	RNA polymerase δ factor RpoE
flhD	flagellar transcriptional activator FlhD	*yahH*	cyclic diguanylatephosphodiesteraseYahH
U12	RNA polymerase δ-factor for flagellar operon protein	*marR*	multiple antibiotic resistance protein MarR
tdcA	Tdc operon transcriptional activator protein TdcA	*U25*	transcriptional defense system activation protein
U13	Threonine dehydratase operon activator protein	*glnK*	nitrogen regulatory protein GlnK
fliZ	flagellar biosynthesis protein FliZ	*hlyU*	transcription activator HlyU
fliT	flagellar protein FliT	*U26*	antitermination protein Q
NAD(P)H	quinoneoxidoreductase, type IV WrbA	*hyaE*	hydrogenase-1 operon protein HyaE
U14	transcription activator, effector binding domain-containing protein	*ydcC*	H repeat-associated protein YdcC
U15	bifunctionalproline dehydrogenase/pyrroline-5-carboxylate dehydrogenase	*rseB*	RseB family protein
cspD	helix-turn-helix domain, RpiRfamily protein	*rpoS*	RNA polymerase δ factor RpoS
csiE	stationary phase-inducible protein CsiE	*U27*	RNase II stability modulator protein
fruR	DNA-binding transcriptional regulator protein FruR	*phoB*	transcriptional regulator protein PhoB
abgR	HTH-type transcriptional regulator protein AbgR	*chaB*	cation transport regulator protein ChaB
U16	making large colonies protein	*U28*	mannitol repressor protein
U17	glucitol operon repressor protein	*pspB*	phage shock protein B PspB
feaR	transcriptional activator protein FeaR	*envY*	porin thermoregulatory protein EnvY
U18	DNA adenine methylase	*rpoH*	alternative δ factor RpoH
hupB	transcriptional regulator protein HU, subunit βHupB	*rpoS*	response regulator RopS
U19	smr domain-containing protein	*ompR*	transcriptional regulatory protein OmpR
iadA	β-aspartyl peptidase IadA	*rscC*	sensor kinase RcsC
flgM	negative regulator of flagella synthesis protein FlgM	*phoR*	phosphate regulon sensor kinase PhoR
cheY	chemotaxis protein CheY	*treR*	trehalose operon repressor protein TreR
cheZ	chemotaxis protein CheZ	*mhpR*	DNA-binding transcriptional

			activator MhpR
evgA	positive transcription regulator EvgA	*bolA*	transcriptional regulator protein BolA
evgS	sensor protein EvgS	*dps*	DNA starvation/stationary phase protection protein Dps
U20	aerotaxis receptor protein	*dnaJ*	chaperone protein DnaJ
rbsK	ribokinaseRbsK	*sfsA*	sugar fermentation stimulation protein SfsA
glc	glc operon transcriptional activator protein	*pspC*	phage shock protein C PspC
U21	Histidine kinase-, DNA gyrase B-, and HSP90-like ATPase	*betI*	transcriptional repressor BetI
malT	HTH-type transcriptional regulator MalT	*U29*	cold-shock DEAD box protein A
ygeV	δ-54-dependent transcriptional regulator YgeV	*hspQ*	Heat shock protein HspQ
galS	mgl repressor and galactoseultrainduction factor GalS	*cgtA*	GTP-binding protein CgtA
hca	hca operon transcriptional activator protein	*fecI*	RNA polymerase δ factor protein FecI
U22	regulatory protein	*pdhR*	transcriptional regulator protein PdhR
cpsI	cold shock-like protein CspI	*holB*	DNA polymerase III, delta subsunitHolB
U23	response regulator protein	*phoP*	transcriptional regulator protein PhoP
U24	colonic acid capsular biosynthesis activation protein A	*phoU*	phosphate transport system regulatory protein PhoU
matA	fimbrillin protein MatA	*cpxR*	transcriptional regulator protein CpxR
cdaR	carbohydrate diacid transcriptional activator CdaR	*virK*	virulence protein VirK
soxS	regulatory protein SoxS		
RNA-associated genes			
U30	RNA polymerase δ factor for flagellar operon protein	*U32*	diguanylatecyclase
rhlE	ATP-dependent RNA helicase RhlE	*rpoH*	Alternative δ factor RpoH
cgtA	GTP-binding protein CgtA	*U33*	poly A polymerase head protein
fecI	RNA polymerase δ factor protein FecI	*truA*	tRNApseudouridine synthase TruA
tilS	tRNA(Ile)-lysidinesynthetaseTilS	*asnA*	aspartate-ammonia ligase AsnA
U31	D-tyrosyl-tRNA(Tyr) deacylase	*cspD*	cold shock domain-containing protein
rpoB	RNA polymerase δ factor RpoS		

159

Supplementary TABLE 5 Induction of genes involved in cell cycle, chemotaxis and pyrimidine nucleotide biosynthetic processes.

Gene	Function	[a]Expression ratios at:		
		0.5 h	1 h	2 h
1. Flagellar assembly				
fliD	Flagellar hook-associated protein	-1.1	-2.0	-2.2
flgB	Flagellar basal-body rod protein FlgB	-6.0	-4.8	-1.3
fliG	Flagellar motor switch protein FliG	-4.4	-2.3	-1.1
fliS	Flagellar protein FliS	-1.7	-1.7	-1.8
flhC	Flagellar transcriptional activator FlhC	-4.6	-3.3	-3.7
flhD	Flagellar transcriptional activator FlhD	-5.0	-4.1	-3.9
fliC	Flagellin	-	-2.6	-4.0
flgM	Negative regulator of flagellin synthesis FlgM	-	-1.1	-1.7
motB	Chemotaxis protein MotB	2.4	2.1	3.2
2. Two-component system				
OmpR family: phosphate assimilation				
phoR	phosphate regulon sensor histidine kinase	1.2	1.0	1.1
phoB	alkaline phosphatase	1.3	1.4	1.6
phoA	alkaline phosphatase	1.0	1.4	-
rstB	sensor histidine kinase RstB	1.3	1.6	2.1
rstA	response regulator RstA	1.7	2.0	2.9
OmpR family: cell envelope protein folding, and protein degradation				
htrA	serine protease Do	4.1	3.4	3.6
OmpR family: multidrug efflux				
mdtA	putative multidrug efflux transporter MdtA	1.5	2.0	2.1
mdtB	RND superfamily, multidrug transport protein MdtB	1.6	1.7	1.6
mdtC	RND superfamily, multidrug transport protein MdtC	1.4	1.5	1.5
OmpR family: flagella regulon				
flhD	flagellar transcriptional activator FlhD	-5.0	-4.1	-3.9

flhC	flagellar transcriptional activator FlhC	-4.6	-3.3	-3.7
fliA	RNA polymerase sigma factor for flagellar operon FliA	-2.9	-2.7	-2.2
fliC	flagellin	-	-2.6	-4.0
motA	chemotaxis protein MotA	-	-1.7	-1.9
NarL family: cps genes				
rcsA	LuxR family transcriptional regulator, capsular biosynthesis positive transcription factor	2.6	2.6	3.7
NtrC family: zinc/lead efflux labile hydrogenase activity				
zraP	zinc resistance-associated protein	1.1	2.2	1.7
Chemotaxis family				
cheR	chemotaxis protein methyltransferaseCheR	1.6	1.1	-1.5
cheB	response regulator CheB	1.6	1.1	-1.5
3. ABC transporters				
Mineraland organic ion transporters				
porG	putrescine transport system ATP-binding protein	2.9	2.3	-
opuBC	osmoprotectant transport system substrate-binding protein	1.9	1.3	-
opuBB	smoprotectant transport system permease protein	1.7	1.5	-
opuBA	osmoprotectant transport system ATP-binding protein	1.5	1.5	-
Phosphate amino acid transporters				
pstS	phosphate transport system substrate-binding protein	1.2	2.0	3.4
pstA	phosphate transport system permease protein	1.1	-	2.1
aapJ, bztA	general L-amino acid transport system substrate-binding protein	1.2	1.9	1.5
fliY	cystine transport system substrate-binding protein	-	1.0	1.3
Peptide and nickel transporters				
dppA	dipeptide transport system substrate-binding protein	1.4	-	-
dppB	dipeptide transport system permease protein	1.6	1.7	-
ABC-2 and other transporters				
yadH	-	1.4	1.2	1.4
Other putative ABC transporters				
yddA	putative ATP-binding cassette transporter	2.1	2.0	1.7
4. Carbohydrate metabolism				
Fructose and mannose metabolism				
E2.4.1.-	fructose and mannose metabolism	1.1	1.9	2.0
gmd, GMDS	GDPmannose 4,6-dehydratase	2.5	2.6	3.6
TSTA3, fcl	GDP-L-fucose synthase	2.2	2.9	3.6
ALDO	fructose-bisphosphatealdolase, class I	2.3	2.2	1.5

GMPP	mannose-1-phosphate guanylyltransferase	-	1.5	3.0
PTS-Fru-EIIA, fruB	PTS system, fructose-specific IIA component	1.6	2.1	-
Pyruvate metabolism				
AldA	lactaldehyde dehydrogenase/glycolaldehyde dehydrogenase	2.4	2.1	1.3
poxB	pyruvate dehydrogenase (quinone)	2.1	1.6	-
ACH1	acetyl-CoA hydrolase	1.2	1.2	-
leuA	2-isopropylmalate synthase	-	1.1	1.6
E1.2.1.10	acetaldehyde dehydrogenase (acetylating)	-	2.1	1.6
Butanoate/alanine, aspartate and glutamate metabolism				
gabD	succinate-semialdehydedehydrogenase/glutarate-semialdehyde dehydrogenase	1.5	1.2	-
ABAT	4-aminobutyrate aminotransferase/(S)-3-amino-2-methylpropionate transaminase	1.4	1.4	-
E1.1.1.-	butanoate metabolism	1.3	1.9	1.9
ttuC, dmlA	tartrate dehydrogenase/decarboxylase/ D-malate dehydrogenase	-	-4.3	-2.7
asnB, ASNS	asparagine synthase (glutamine-hydrolysing)	1.3	1.8	-
asnA	aspartate--ammonia ligase	2.0	1.5	-
glsA, GLS	glutaminase	1.0	2.9	-
Methane metabolism				
frmA, ADH5, adhC	S-(hydroxymethyl)glutathione dehydrogenase/ alcohol dehydrogenase	1.4	2.0	-
katE, CAT, catB, srpA	Catalase	3.1	2.4	2.1
Arginine and proline metabolism				
glsA, GLS	glutaminase	1.0	2.9	-
prr	aminobutyraldehyde dehydrogenase	1.2	1.8	-
astC	succinylornithine aminotransferase	-	1.4	1.1
Glycolysis/Gluconeogenesis				
ALDO	fructose-bisphosphatealdolase, class I	2.3	2.2	1.5
GAPDH, gapA	glyceraldehyde 3-phosphate dehydrogenase	1.4	1.8	-
ADH1_7	alcohol dehydrogenase 1/7	1.4	2.0	1.6
Galactose metabolism				
UGP2, galU, galF	UTP--glucose-1-phosphate uridylyltransferase	1.4	1.3	1.3
Glyoxylate and dicarboxylate metabolism				
aldA	Lactaldehyde dehydrogenase/ glycolaldehyde dehydrogenase	2.4	2.1	1.3
katE, CAT, catB, srpA	Catalase	3.1	2.4	2.1
ttuC, dmlA	tartrate dehydrogenase/decarboxylase/D-malate dehydrogenase	-	-4.3	-2.7
5.Nucleotide metabolism				
Pyrimidinemetabolism				

rutB	Ureidoacrylateperacid hydrolase	1.4	1.3	2.6
cpdB	2',3'-cyclic-nucleotide 2'-phosphodiesterase/3'-nucleotidase	1.5	2.0	-1.0
Purine metabolism				
cpdB	2',3'-cyclic-nucleotide 2'-phosphodiesterase / 3'-nucleotidase	1.5	2.0	-1.0
XDH	xanthine dehydrogenase/oxidase	2.5	2.7	1.8
6. Cell cycle				
pleD	cell cycle response regulator	1.4	1.2	1.3
dgcB	Diguanylatecyclase	-	1.2	1.9
mmpA(*rseP*)	Regulator of sigma E protease	1.1	-	-
7. RNA degradation				
rhlE	ATP-dependent RNA helicase RhlE	-	-	1.2
cshA	ATP-dependent RNA helicase DeaD	-	-	1.7
8. Peptidoglycan biosynthesis				
bacA	Undecaprenyl-diphosphatase	1.8	1.5	2.1
dacC, dacA, dacD	D-alanyl-D-alanine carboxypeptidase, penicillin-binding protein	1.1	1.3	1.3
mrcA/B	Penicillin-binding protein	1.2	1.1	1.4

162

Supplementary TABLE 6 Marker genes of the *E. coli* transcript response library treated with arenicin-3 and colistin (S9).

Symbol	Description
Arenicin-3	
Genes involved in translation	
rpl	50S ribosomal proteins
rps	50S ribosomal proteins
rpm	30S ribosomal proteins
Translation factors	Initiation, elongation, and termination
PSP genes	
pspA	phage shock protein, inner membrane protein
pspB	phage shock protein B
pspC	phage shock protein C
pspD	phage shock protein D
Genes upregulated	
osmB	lipoprotein, osmotically inducible
bdm	biofilm-dependent modulation protein
soxS	regulation of superoxide response regulon
Colistin	
Osmotic shock genes	
osmB	lipoprotein, osmotically inducible
osmC	proxiredoxin
osmY	smotically-inducible protein Y precursoror
osmE	osmotically-inducible lipoprotein E precursor
bdm	biofilm-dependent modulation proteinosmotically inducible
sra	rpsV, osmotically inducible
rcsA	positive regulator for ctr capsule biosynthesis

Supplementary References:

1. **Hancock RE.** 1984. Alterations in outer membrane permeability. Ann Rev Microbiol **38**:237–264.

2. **Zhang Y, Teng D, Wang X, Mao R, Cao X, Hu X, Zong L, Wang J.** 2015. In vitro and in vivo

characterization of a new recombinant antimicrobial peptide, MP1102, against methicillin-resistant Staphylococcus aureus. Appl Microbiol Biotechnol **99**:6255–6266.

3. **Jiao J, Mao RY, Wang XM, Zhang Y, Teng D, Feng XJ, Wang JH.** 2015. GAP-initiated constitutive expression of a novel plectasin-derived peptide MP1106 by *Pichia pastoris* and its activity against *Streptococcus suis*. Process Biochem **50**:253–261.
4. **Ling LL, Schneider T, Peoples AJ, Spoering AL, Engels I, Conlon BP, Mueller A, Schäberle TF, Hughes DE, Epstein S, Jones M, Lazarides L, Steadman VA, Cohen DR, Felix CR, Fetterman KA, Millett WP, Nitti AG, Zullo AM, Chen C, Lewis K.** 2015. A new antibiotic kills pathogens without detectable resistance. Nature **517**:455–459.
5. **Ouberai M, El Garch F, Bussiere A, Riou M, Alsteens D, Lins L, Baussanne I, Dufrêne YF, Brasseur R, Decout JL, Mingeot-Leclercq MP.** 2011. The *Pseudomonas aeruginosa* membranes: a target for a new amphiphilic aminoglycoside derivative? Biochim Biophys Acta **1808**:1716–1727.
6. **Falla TJ, Karunaratne DN, Hancock RE.** 1996. Mode of action of the antimicrobial peptide indolicidin. J Biol Chem **271**:19298–19303.
7. **Wang X, Wang X, Teng D, Zhang Y, Mao R, Xi D, Wang J.** 2014. Candidacidal mechanism of the arenicin-3-derived peptide NZ17074 from *Arenicola marina*. Appl Microbiol Biotechnol **98**:7387–7398.
8. **Kozlowska J, Vermeer LS, Rogers GB, Rehnnuma N, Amos SB, Koller G, McArthur M, Bruce KD, Mason AJ.** 2014. Combined systems approaches reveal highly plastic responses to antimicrobial peptide challenge in *Escherichia coli*. PLoS Pathog **10**:e1004104.
9. **Nielsen AK, Sandvang D, Neve S, Kruse T, Kristensen H-H.** Transcriptional profiling indicates

a dual mode-of-action of Arenicin-3. Novozymes A/S, Bagsvaerd, Denmark. Poster No.F1-2072.
